# Targeting NOTCH-immune crosstalk in gliomas: from mechanisms to therapeutics

**DOI:** 10.3389/fonc.2026.1936605

**Published:** 2026-10-07

**Authors:** Safieh Ebrahimi, NoorMohammad Meshkinkhood, Ali Aliee, Li Yuhan, Maryam Koulivand, Zahra Koulivand, Ali Gorji

**Affiliations:** 1 Bioinformatics Research Center, Basic Sciences Research Institute, Mashhad University of Medical Sciences, Mashhad, Iran; 2 Shefa Neuroscience Research Center, Khatam Alanbia Hospital, Tehran, Iran; 3 Department of Clinical Biochemistry, Faculty of Medicine, Mashhad University of Medical Sciences, Mashhad, Iran; 4 Department of Clinical Biochemistry, Kermanshah University of Medical Sciences, Kermanshah, Iran; 5 Department of Neurosurgery, Xinhua Hospital Affiliated to Shanghai Jiao Tong University School of Medicine, Shanghai, China; 6 Epilepsy Research Center, Department of Neurosurgery, Münster University, Münster, Germany; 7 Neuroscience Research Center, Mashhad University of Medical Sciences, Mashhad, Iran

**Keywords:** epigenetic regulation, immune modulation, stem cells, therapeutic resistance, tumor micromilieu

## Abstract

Glioblastoma (GBM) is a highly aggressive and treatment-resistant malignancy characterized by an immunosuppressive tumor microenvironment (TME) that promotes disease progression and poor clinical outcomes. Increasing evidence suggests that NOTCH signaling is a key regulator of GBM progression, governing both tumor cell plasticity and immune evasion. In addition to its roles in development and stem cell maintenance, NOTCH signaling modulates interactions among glioma stem-like cells, immune cells, and stromal components. NOTCH signaling in both tumor and stromal compartments modulates immune cell fate decisions, antigen presentation, and inflammatory cytokine expression. This suggests its potential role as a key modulator of immune crosstalk within the TME of GBM. Moreover, dysregulation of the NOTCH signaling pathway may lead to an immunosuppressive TME, enhance tumor heterogeneity, and restrict responses to conventional therapies. Recent investigations also reveal the epigenetic and non-coding RNA patterns that reinforce aberrant NOTCH activity in GBM. Understanding the multifaceted interactions among NOTCH signaling, oncogenic pathways, and immunomodulatory mechanisms could provide insights into the molecular pathogenesis of GBM progression. Here, we discuss the pleiotropic and context-dependent functions of NOTCH signaling in GBM, with particular emphasis on its implications for tumor–immune interactions and its potential as a therapeutic target to address immune escape and treatment resistance.

## Introduction

Gliomas represent a diverse category of primary brain tumors defined according to the classification system of the World Health Organization into various grades and histological subtypes, including astrocytoma (isocitrate dehydrogenase (IDH)-mutant, grades 2–4), oligodendroglioma (IDH-mutant and 1p/19q-codeleted), and glioblastoma (GBM, IDH-wildtype, grade 4) (1–3). GBM is the most aggressive and lethal variant, constituting over 50% of diffuse gliomas and demonstrating a median survival time of only 12 to 15 months, even with intensive multimodal therapies (4, 5). The dismal prognosis of GBM is attributed not only to the intrinsic properties of tumor cells, such as cellular diversity, rapid growth, widespread infiltration, genomic instability, and therapy resistance, but also to the intricate and immunosuppressive tumor microenvironment (TME) (4–6). TME is an intricate and interconnected network system of cancer cells, cancer stem-like cells, reactive astrocytes, immune cell infiltrates (including myeloid-derived suppressor cells (MDSCs), microglia and tumor-associated macrophages (TAMs), and T cells), fibroblasts, and blood vessels, along with an extracellular matrix rich in growth factors, chemokines, and cytokines that all of which collectively influence tumor progression, immune evasion, and resistance to therapy (7–9). Within this micromilieu, complex intercellular signaling networks regulate immune evasion, angiogenesis, and tumor invasion. Among the key signaling pathways mediating communication within the TME, NOTCH signaling serves a dual function, acting as both an oncogenic driver and a tumor suppressor, depending on cellular context (10, 11). The NOTCH signaling pathway is a conserved evolutionary pathway that mediates intercellular communication and plays pivotal roles in stem cell preservation, cell fate determination, and tissue equilibrium. In GBM, aberrant activation of NOTCH receptors (NOTCH 1–4) and their ligands (Jagged (JAG) 1 and 2, as well as Delta-like ligands (DLL) 1, 3, and 4) has been implicated in maintaining the cancer stem cell (CSC) phenotype, promoting resistance to therapy, and driving tumor invasion. The four mammalian NOTCH receptors (NOTCH 1–4) and their canonical ligands (DLL1, DLL3, DLL4, JAG1, and JAG2) are often overexpressed in glioma tissues, and their activation leads to the nuclear translocation of the NOTCH intracellular domain (NICD) that triggers the transcription of oncogenic target genes, like hairy and enhancer of split (Hes) 1 and Hey1. The NOTCH pathway is particularly crucial for the cellular maintenance and viability of glioma stem-like cells (GSCs), a subpopulation that drives tumor recurrence and therapeutic resistance (12–14). Enhanced expression of NOTCH ligands, including DLL4 and JAG1, has been identified in both tumor cells and the GBM vasculature, contributing to angiogenesis and stemness preservation (15–17). More recently, NOTCH3 has been identified as a clinically significant subtype-specific oncogenic factor. Elevated NOTCH3 expression correlates with poorer overall survival, enhanced tumor cell proliferation, and greater immune cell infiltration in IDH1-wildtype, classical, and mesenchymal GBM subtypes (17). Beyond its intrinsic effects on tumor cells, NOTCH signaling regulates the immune signature of the glioma TME, showing dual functions, with either pro-inflammatory or anti-inflammatory effects depending on the specific cellular context and signaling strength (18). Within myeloid cells, the activation of NOTCH signaling can strengthen immunosuppression, whereas in T cells, it has the potential to promote differentiation and effector responses under particular conditions (19–21). This dual and context-sensitive function of NOTCH signaling presents both challenges and opportunities for designing novel therapeutic strategies, making it a potential target to overcome immunosuppression and enhance therapeutic efficacy in glioma. However, the context-sensitive effects of NOTCH activation point out the necessity for precise modulation to limit interference with anti-cancer immunity. Gaining a comprehensive understanding of the multifaceted role of NOTCH in the immune microenvironment of GBM is required for the rational design of NOTCH-targeted therapies and their incorporation into immunotherapeutic approaches. Given these considerations, there is an increasing necessity to more accurately define the function of NOTCH signaling in shaping the immunosuppressive microenvironment of GBM. While existing literature has thoroughly characterized the tumor-intrinsic oncogenic functions of NOTCH, particularly in gliomagenesis, stemness, lineage plasticity, and therapeutic resistance (22–27), the immune-related aspect of NOTCH signaling is still not well understood and has not been clearly brought together across studies. Therefore, in this review paper, we highlight the role of NOTCH signaling in shaping the TBM of GBM, with a particular focus on its context-dependent effects on immune regulation. Specifically, we discuss how NOTCH signaling affects key immune cell populations, including microglia, TAMs, MDSCs, and T-cell subsets, as well as its impact on cytokine networks, metabolic reprogramming, and intercellular communication within the TME. Our objective is to integrate current mechanistic insights across tumor and stromal compartments, highlighting how NOTCH signaling contributes to immune modulation and, in many contexts, immune suppression and tumor immune evasion. Importantly, we critically assess both supportive and contradictory evidence to clarify the context-dependent nature of NOTCH activity in GBM. By synthesizing these findings, this article aims to address a key gap in the field: the lack of a comprehensive framework linking NOTCH signaling to tumor–immune crosstalk in GBM. Finally, we also address the therapeutic implications of targeting NOTCH signaling, emphasizing the need for precise, cell–type–specific, and context-aware strategies to effectively incorporate NOTCH modulation into immunotherapeutic approaches.

## Activation and signal transduction mechanisms of NOTCH pathway

NOTCH Signaling is triggered by a precisely controlled proteolytic cascade that begins with the interactions between NOTCH receptors (NOTCH 1-4) and their respective signaling ligands (JAG1, JAG2, as well as Dll1, Dll3, and Dll4) expressed on adjacent cells (28–31). NOTCH receptors are produced as single-pass transmembrane precursors within the endoplasmic reticulum. After being translocated to the Golgi apparatus, they experience furin-mediated cleavage (S1 cleavage), resulting in a heterodimeric receptor that comprises an extracellular subunit (NECD) and a transmembrane subunit (NTM). The fully developed receptor is then transported to the plasma membrane, where it is ready for ligand interaction. The activation of NOTCH signaling occurs when the binding of a ligand to its receptor causes conformational alterations in the NOTCH extracellular domain. This interaction necessitates the mechanical force produced by the endocytosis of the ligand-receptor complex within the signaling cell, leading to sequential proteolytic cleavage events: *(i)* S2 cleavage by ADAM (a disintegrin and metalloproteinase), which removes the extracellular domain, and *(ii)* S3 cleavage by γ-secretase, which releases the NOTCH intracellular domain (NICD) from the membrane (28–31).

The released NICD migrates to the nucleus, where it establishes a transcriptional activation complex in conjunction with the recombination signal-binding protein for immunoglobulin kappa J region (RBP-J, alternatively termed CSL/CBF1) and the coactivator Mastermind-like (MAML). This assembly facilitates the expression of primary NOTCH target genes, which encompass members of the Hes (Hes1, Hes5) and Hey (Hey1, Hey2) families, implicated in regulating cell differentiation, proliferation, and viability (29, 32, 33). The core components and activation steps of the NOTCH pathway are illustrated in **Figure 1**. Beyond the canonical RBP-J-dependent pathway, NOTCH signaling exerts diverse cellular effects through non-canonical pathways that are increasingly recognized for their regulatory roles in lineage-specific differentiation, progenitor cell function, and tumorigenesis (34). In these alternative pathways, the NICD bypasses the classical transcriptional activation via RBP-Jκ and instead interacts directly with a range of oncogenic pathways (35–37). Importantly, NOTCH can regulate Wnt/β-catenin signaling, either by stabilizing β-catenin or controlling Wnt receptor expression (38, 39). Furthermore, crosstalk with the JAK/STAT axis has been observed, where NOTCH activates IL-6/STAT3 signaling through a mechanism regulated by the NF-κB components IKKα/IKKβ, pointing to the role of NOTCH in cancer-associated inflammation (40). The PI3K/AKT/mTOR pathway is another critical effector of non-canonical NOTCH function. For instance, NICD interacts with mTORC2 to activate AKT, which is critical for the maintenance of brain tumor–initiating cells (41–43). A key advancement in understanding NOTCH signaling in GBM requires distinguishing the roles of individual receptors and ligands, as growing evidence shows that this pathway exhibits considerable functional heterogeneity. Quantitative analyses demonstrate that although core components are coordinately upregulated in GBM, the four mammalian receptors (NOTCH1–4) drive divergent or even opposing phenotypes (32). NOTCH1, for example, exhibits a context-dependent and somewhat paradoxical profile. Although higher NOTCH1 expression has been reported in patients with longer survival (>1 year), its overexpression has also been accompanied by reduced overall survival, indicating a dual role in gliomagenesis (44, 45). Furthermore, NOTCH1 is preferentially expressed in peritumoral glioma stem cells, indicating spatial heterogeneity in receptor activity (32, 46). NOTCH4 expression shows a clear association with increasing tumor grade and is preferentially elevated in primary GBM, consistent with a role in tumor progression and aggressive disease phenotypes (47). NOTCH3 acts as a consistent pro-tumorigenic oncogene enriched in aggressive classical and mesenchymal subtypes, correlating with poor survival and high immune infiltration (48, 49). By contrast, NOTCH2 supports neural stem cell maintenance and astroglial lineage entry but negatively correlates with oligodendroglial lineage commitment (47, 50).

**Figure 1:**
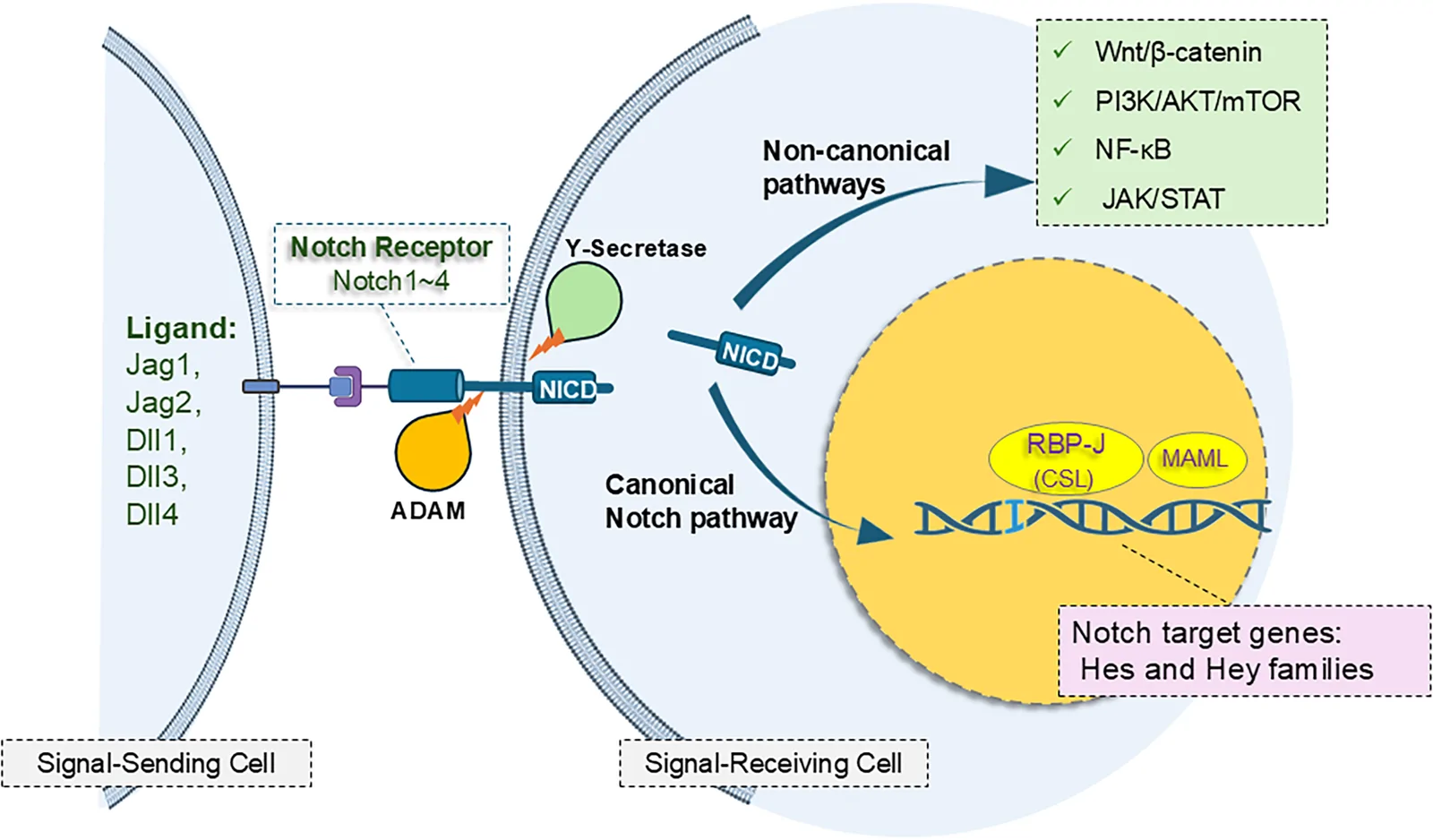
Molecular architecture of canonical and non-canonical NOTCH signaling transduction. Activation is initiated by ligand–receptor interactions (JAG1/2, DLL1/3/4 engaging NOTCH1–4) between adjacent signal-sending and receiving cells. This interaction triggers sequential proteolytic events: S2 cleavage by ADAM metalloproteinases and subsequent S3 cleavage by the γ-secretase complex, releasing the active NOTCH intracellular domain (NICD). Canonical Pathway: NICD translocates to the nucleus, associates with the DNA-binding protein RBP-J (CSL) and co-activator MAML, displacing co-repressors to drive transcription of target genes (Hes and Hey families). Non-canonical Pathway: NICD bypasses RBP-J to crosstalk directly with alternative oncogenic cascades (including Wnt/β-catenin, PI3K/Akt/mTOR, NF-κB, and JAK/STAT), coordinating diverse cellular responses.

Moreover, canonical ligands trigger distinct and often antagonistic programs. In the vascular compartment, DLL4 and JAG1 exert opposing effects on angiogenic sprouting. DLL4, predominantly expressed by endothelial tip cells, inhibits excessive sprouting, whereas JAG1, expressed in stalk cells, counteracts DLL4-mediated signaling and promotes vascular sprouting (32, 51–53). Furthermore, DLL3 acts primarily as a cis-inhibitor; it is epigenetically regulated via promoter methylation and is highly upregulated in IDH1-mutant gliomas, where it correlates with improved overall survival. Conversely, JAG1 serves as a major driver of local and systemic immune evasion, directly upregulating PD-L1 via the PI3K/Akt pathway and promoting regulatory T cell (Treg) recruitment and systemic lymphopenia (23). **Table 1** presents the distinct NOTCH receptors and their canonical ligands, detailing their expression patterns, cellular contexts, functional effects, and clinical implications in glioma.

**Table 1:** Receptor- and ligand-specific functions of notch signaling in glioblastoma (GBM).

NOTCH receptor/ligand	Cell types	Effects in GBM/Glioma	Clinical implications	Ref.
*NOTCH1*	Peritumoral GSCs, bulk tumor cells, Thymic/systemic T cells	Sustains GSC self-renewal; Drives radio/chemoresistance; mediates PDL1 expression and immune suppression	Dual prognostic role; target for receptor-specific antibody therapies	(32, 44)
*NOTCH2*	GSCs, neurospheres, oligodendroglioma cells	Maintains stemness and promotes astrocytic differentiation; limits GBM neurosphere proliferation.	Tumor-promoting in GBM but suppressive in oligodendroglioma/ LGG	(50, 54)
*NOTCH3*	GBM cells (classical/mesenchymal), CD4+ T cells	Promotes cell proliferation, invasion, and high immunosuppressive immune cell infiltration	Independent prognostic biomarker; candidate therapeutic and diagnostic target	(17, 48, 49)
*NOTCH4*	Primary GBM tumor cells, Tumor vascular endothelial cells	Cooperates with DLL4 in angiogenesis; wild-type is proangiogenic.	Loss-of-function mutations serve as a predictive biomarker for favorable ICI therapy response	(47, 52, 55)
*DLL1*	Bulk GBM tumor tissue (co-upregulated with NOTCH1/NOTCH4/JAG1)	Activates PI3K/AKT signaling to promote cell survival and tumor angiogenesis.	Marker for radiotherapy resistance; Component of a pro-tumorigenic signature	(32, 52, 53, 56)
*DLL3*	IDH1-mutant glioma/LGG tumor cells	Acts as a noncanonical inhibitory ligand; epigenetically regulated via promoter methylation.	Subtype-specific prognostic biomarker in IDH- mutant LGG; candidate for stratification	(57–59)
*DLL4*	Endothelial tip cells (tumor vasculature); Tumor cells	Restrains excessive angiogenic sprouting (VEGF feedback); drives anti-VEGF resistance.	Poor-prognosis marker; therapeutic target to disrupt vascular-immune niche	(23, 52, 60)
*JAG1*	Vascular stalk cells, LGG/GBM tumor cells, TAMs, Thymic epithelial cells	Antagonizes DLL4 to promote sprouting; drives TAM-M2- polarization and systemic thymic atrophy.	Key driver of microenvironmental immune escape; candidate combination target with ICIs	(23, 61, 62)
*JAG2*	Not distinctly characterized for specific glioma/GBM cell populations	One of the canonical NOTCH ligands in general pathway-activation and receptor–ligand interaction contexts, No GBM data were identified for JAG2	Represents a knowledge gap warranting investigation of JAG2-specific effects and clinical correlates in glioma	(10, 11)

GBM, glioblastoma; LGG, low-grade glioma; GSC, glioma stem-like cell; TAM, tumor-associated macrophage; PD-L1, programmed death-ligand 1; ICI, immune checkpoint inhibitor; VEGF, vascular endothelial growth factor.

## Physiological and pathological significance of NOTCH signaling pathway in GBM

NOTCH signaling pathway is important for processes such as maintenance of stem cell populations, cell fate specification, and lineage determination during embryogenesis, as well as for regulating tissue homeostasis. In the central nervous system, NOTCH enables neurogenesis and gliogenesis by modulating the balance between progenitor cell proliferation and differentiation (63). It is also central in shaping immune cell development, especially in T cell lineage specification and myeloid differentiation (64). However, dysregulation of NOTCH signaling can contribute to a wide range of pathological conditions. Abnormal NOTCH activity resulting from genetic mutations (65), epigenetic changes (66), or imbalanced ligand-receptor expression (67) has been implicated in congenital disorders (68), fibrotic diseases (69), and multiple cancer types (37). In hematologic malignancies such as T-cell acute lymphoblastic leukemia, activation of NOTCH1 mutations promotes uncontrolled cell proliferation (70), whereas in epithelial cancers such as squamous cell carcinoma, NOTCH typically acts as a tumor suppressor (71). In the context of GBM, NOTCH signaling contributes to different hallmarks of gliomagenesis, many of which are outlined in **Figure 2**. High-throughput transcriptomic studies and tissue profiling have shown increased expression of NOTCH receptors (NOTCH1–4), canonical ligands (Jagged1, DLL1, DLL4), and downstream effectors (Hes1, Hey1) in GBM tissues compared to control brain tissue (12, 54, 72, 73). NOTCH1, in particular, is often upregulated and is associated with a higher glioma grade, poor prognosis, and greater invasiveness (44, 74). Emerging evidence highlights the crucial role of NOTCH3 in the pathogenesis and clinical behavior of GBM. While all four NOTCH receptors (NOTCH1–4) are upregulated in GBM, NOTCH3, in particular, has been identified as an independent prognostic marker associated with poor survival outcomes (17). Integrated analyses of TCGA and CGGA datasets revealed that high NOTCH3 expression is enriched in aggressive GBM subtypes, including the classical and mesenchymal types, and correlates with IDH1 wild-type and non-G-CIMP tumors, which are known to have a poor prognosis (17). NOTCH3 expression also demonstrated a strong association with tumor cell proliferation and immune cell infiltration, particularly involving CD4^+^ T cells, macrophages, and dendritic cells (75). Immunohistochemical validation in a clinical GBM cohort further confirmed that over 50% of patient samples exhibited high NOTCH3 protein levels, and patients with elevated NOTCH3 expression had significantly shorter survival times. One of the most significant functions of NOTCH is in sustaining the GSC population (76). Activation of NOTCH1 and its downstream targets, such as Hes1 and Hey1, maintains GSC self-renewal, suppresses differentiation, and confers resistance to radiation and chemotherapy (12, 77). Moreover, NOTCH signaling interacts synergistically with key oncogenic pathways, such as PI3K/Akt, STAT3, and NF-κB, to enhance gliomagenesis, particularly by facilitating mesenchymal-like transcriptional transition (MES-like state) and sustaining angiogenesis (73, 78, 79). Under hypoxic stress, hypoxia-inducible factor (HIF)-1α cooperates with NOTCH to preserve the GSC phenotype and promote a vascular, pro-invasive tumor architecture (80). Moreover, NOTCH activity is affected by epigenetic regulators and miRNAs. For instance, miR-34a, miR-181c, and histone methylation patterns affect the expression of NOTCH effectors like Hes1 and Hey1, thereby linking chromatin dynamics to NOTCH-mediated oncogenesis (81–83). The enhanced cell proliferation induced by knockdown of KMT2A, an epigenetic regulator and histone methyltransferase, could be reversed by expressing either constitutively active NOTCH1 or NOTCH3 in GBM (84). The function of the NOTCH signaling pathway in glioma is highly context-dependent. While hyperactivation of NOTCH signaling generally promotes tumorigenesis, glioma subtypes exhibit distinct patterns of pathway alteration. For instance, DLL3 expression is more prevalent in IDH-mutant gliomas, whereas in mesenchymal GBM, NOTCH activation is enriched in patients with high p-STAT3 expression, suggesting a transcriptional convergence between NOTCH and STAT3 signaling (85, 86). These observations highlight the marked heterogeneity of NOTCH pathway regulation across glioma subtypes and suggest that its downstream effects are shaped by the surrounding molecular and transcriptional landscape. Consistent with this subtype-specific complexity, NOTCH signaling is widely recognized as a highly context-dependent pathway, capable of exerting both oncogenic and tumor-suppressive functions depending on the cellular and molecular environment. While this review primarily focuses on its pro-tumorigenic and immunomodulatory roles in GBM, accumulating evidence highlights that NOTCH may also function as a tumor suppressor in specific glioma contexts (11). Indeed, comprehensive genomic analyses of lower-grade gliomas (LGGs) have identified recurrent loss-of-function mutations in key NOTCH pathway components, including NOTCH1, NOTCH2, and RBPJ, which are associated with reduced expression of canonical target genes and increased tumor cell proliferation, suggesting that pathway inactivation contributes to glioma progression (87–91). Consistently, experimental models demonstrate that genetic ablation of NOTCH signaling accelerates tumor growth, whereas enforced activation of NOTCH2 or downstream effectors such as HEY2 can suppress glioma cell proliferation and tumor formation (92, 93). Mechanistically, these tumor-suppressive effects have been linked, at least in part, to NOTCH-mediated repression of pro-neurogenic and proliferative drivers such as ASCL1, thereby maintaining cellular quiescence and blocking malignant transformation (92, 94–96). Importantly, such findings underscore that the functional outcome of NOTCH signaling is shaped by lineage state, genetic background (e.g., IDH mutation status), and tumor subtype, which may explain the apparent discrepancies across studies. Therefore, a critical interpretation of NOTCH biology in GBM requires integrating these context-specific and sometimes opposing roles, particularly when considering therapeutic strategies aimed at modulating this pathway.

**Figure 2:**
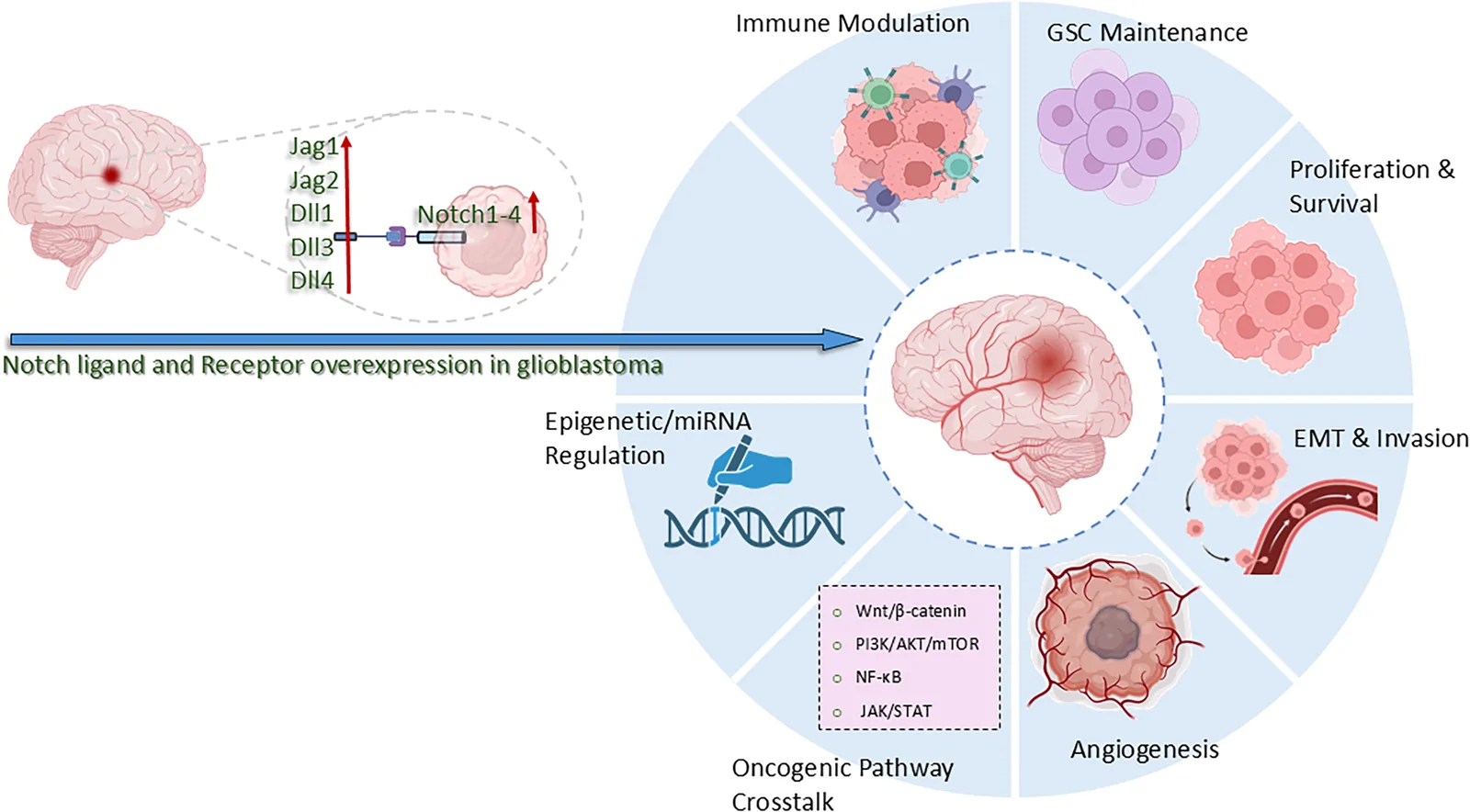
NOTCH signaling–mediated oncogenic mechanisms in GBM. NOTCH signaling acts as a central regulator of gliomagenesis, contributing to multiple malignant processes. This schematic illustrates key oncogenic functions radiating from a central NOTCH signaling hub, including GSC maintenance, enhanced proliferation and survival, MES-like state and invasion, angiogenesis, interaction with other oncogenic pathways (e.g., PI3K/Akt, STAT3), epigenetic and miRNA-mediated regulation, and modulation of the tumor immune microenvironment.

Beyond its tumor-intrinsic functions, the NOTCH signaling pathway also contributes to the regulation of the glioma TME (97). In particular, NOTCH signaling modulates immune cell differentiation, cytokine production, and angiogenic signaling within the TME (11, 98, 99). However, these effects are highly context-dependent and require distinction between tumor cell–intrinsic and microenvironmental mechanisms. Emerging evidence supports a dual mode of action. On one hand, activation of NOTCH signaling within glioma cells and GSCs can regulate the expression of immunomodulatory factors, including cytokines such as IL-10 and TGF-β and immune checkpoint molecules, thereby indirectly shaping immune responses and promoting immunosuppressive phenotypes in TAMs and T cells. On the other hand, NOTCH signaling can also operate in a cell-autonomous manner within immune cells. For example, NOTCH activation in myeloid cells, including macrophages and microglia, influences polarization programs through interactions with NF-κB and STAT signaling pathways, while in T cells it contributes to lineage specification and effector differentiation (64). In GBM, NOTCH receptors are primarily expressed on tumor cells, whereas their ligands are predominantly expressed on endothelial cells (100). Endothelial cells in the perivascular niche express NOTCH ligands that support GSC self-renewal (101), while stromal cells and TAMs engage NOTCH to support immunosuppressive circuits (102). Collectively, the Notch pathway plays a dual role as both a driver of glioma pathogenesis and a key orchestrator of immune regulation within the TME. Importantly, much of the current evidence is derived from preclinical models or correlative analyses, and the extent to which these interactions represent causative, cell–type–specific mechanisms in human GBM remains incompletely defined. Its diverse biological effects, including stemness maintenance, angiogenesis, and immune regulation, highlight its potential as a therapeutic target, as discussed in the following section.

## Therapeutic targeting of NOTCH signaling pathway in GBM

Targeting NOTCH signaling is a potential therapeutic strategy for GBM, with several approaches evaluated in preclinical studies and early clinical trials (103). γ-secretase inhibitors (GSIs), such as MRK003, DAPT, and RO4929097, are the most widely studied NOTCH inhibitors. Preclinical studies have shown that these agents suppress GSC proliferation, reduce stemness markers (CD133, Nestin, BMI-1, OLIG2), and sensitize tumors to radiation and temozolomide (TMZ) by downregulating AKT and STAT3 pathways (104–109). MRK003, for instance, effectively reduced tumor size and enhanced radiosensitivity in CD44 high/CD133 low GBM-initiating cells, marking it as a potential candidate for stem-targeted therapy (108). Similarly, RO4929097, another GSI, exhibited anti-proliferative effects in GBM spheroid models and prolonged survival in orthotopic mouse models, especially when combined with radiation and chemotherapy (107, 109). DLL4-induced NOTCH signaling promotes resistance to anti-vascular endothelial growth factor (VEGF) therapy by driving large-vessel formation and activating alternative pathways, and blocking these pathways enhances the efficacy of bevacizumab, an anti-VEGF drug used in GBM, in DLL4-expressing tumors in xenograft GBM mouse models (60). Silencing of EFEMP1, an extracellular matrix protein, reduces GBM cell survival in response to TMZ, whereas its overexpression promotes TMZ resistance through multiple signaling pathways, including γ-secretase–dependent activation of NOTCH signaling (110). Combining NOTCH inhibition with radiation markedly reduced both proliferation and self-renewal in three-dimensional organotypic explant cultures derived from surgical GBM specimens (105, 109). GBM neurosphere cultures with high endogenous NOTCH activity were sensitive to NOTCH inhibition, showing increased G0/G1 arrest and reduced colony formation with limited impact on self-renewal or differentiation, whereas NOTCH activation decreased the G0/G1 population, enhanced colony formation, self-renewal, and differentiation (106). Inhibition of the NOTCH signaling pathway via DN-MAML1 expression or application of MRK003 (a GSI) significantly reduced GBM cell growth both *in vitro* and *in vivo*, with NOTCH1 and NOTCH2 contributing differentially. NOTCH2 plays a predominant role and suppresses the proliferation of human GBM-derived neurospheres (104).

Despite these encouraging experimental findings, clinical translation remains limited. A Phase 0/I trial of RO4929097 in newly diagnosed GBM patients showed a reduction in NICD levels and CD133^+^ GSCs, indicating on-target activity, but limited tumor control potentially due to drug metabolism issues (CYP3A4 auto-induction) and NOTCH pathway redundancy (111). Similarly, a Phase I study of combination therapy with RO4929097 and bevacizumab showed tolerability and partial responses in a few patients, but with limited long-term benefit (112). In a phase II study, treatment with the γ-secretase inhibitor RO4929097 in 47 patients with recurrent GBM has shown minimal clinical activity, achieving a 6-month progression-free survival of 4% and inhibiting neurosphere formation in only 1 of 7 resected tumor samples (113). The clinical utility of RO4929097 in GBM is limited by several factors, including poor bioavailability across the blood-brain barrier, variable efficacy between tumor grades due to differential NOTCH pathway activity, and off-target toxicity implicating normal physiological functions (114). To overcome these challenges, CB-103, a first-in-class small molecule that blocks the NOTCH transcriptional activation complex, is under evaluation in an ongoing Phase I/IIa trial, testing its effectiveness both as a monotherapy and in combination with other therapies (115). Unlike GSIs, CB-103 bypasses upstream receptor processing, is well tolerated, and may avoid the toxicity issues associated with γ-secretase inhibitors (116). Alternative approaches involve NOTCH receptor–specific antibodies, which minimize the toxicity associated with pan-NOTCH inhibition. For instance, selective NOTCH1 blockade preserves antitumor efficacy while decreasing gastrointestinal adverse effects (117). Another potential approach targets α-secretase, the enzyme responsible for the initial proteolytic step that precedes γ-secretase–mediated NOTCH activation. In this context, Floyd et al. demonstrated that INCB3619, a dual ADAM10/17 inhibitor, effectively suppresses GSC growth and tumor progression *in vivo*, significantly prolonging survival in mouse models of GBM (118). The antitumor effect was attributed to diminished NOTCH signaling activity, as evidenced by decreased expression of the NOTCH downstream effectors Hes1 and Hey1. Moreover, combinatorial treatment with DAPT, a γ-secretase inhibitor, further enhanced these effects, not only inhibiting classical NOTCH target genes but also downregulating glioma-associated oncogenic mediators, such as leukemia inhibitory factor and YKL-40 (CHI3L1) (118). While NOTCH inhibitor monotherapy has shown limited clinical efficacy, combination approaches, particularly with radiotherapy, TMZ, or immune-modulating agents, may provide greater translational potential (119). A detailed overview of current and emerging therapeutic strategies targeting the NOTCH pathway in GBM is provided in **Table 2**. Although **Table 2** summarizes current therapeutic strategies targeting the NOTCH signaling pathway in glioma, several limitations should be acknowledged. First, the available clinical evidence remains limited, as most studies are derived from early-phase clinical trials with relatively small patient cohorts, which restricts the generalizability of the reported outcomes (115, 120, 121). In addition, heterogeneity among patient populations, including differences between GBM and anaplastic astrocytoma, as well as variations in molecular characteristics such as IDH1/2 mutation status, may influence treatment response and complicate the interpretation of clinical results (122). Another important limitation relates to challenges in evaluating treatment response, since radiological progression during therapy may represent pseudoprogression caused by therapy–induced blood–brain barrier disruption rather than true tumor progression (121, 123). Furthermore, pharmacokinetic variability and heterogeneous drug penetration across tumor regions due to the blood–brain barrier can affect the extent of NOTCH pathway inhibition achieved *in vivo* (111). Moreover, the context-dependent nature of NOTCH signaling in glioma, where both activation and suppression can differentially influence tumor and immune compartments, further complicates therapeutic targeting and may contribute to heterogeneous treatment responses. These observations underscore important considerations for the development of future NOTCH-directed therapeutic strategies. In this context, a deeper understanding of the isoform-specific and context-dependent roles of NOTCH signaling, as well as its interactions with immune and stromal components of the TME, will be critical for the development of precision-based therapies for GBM. Notably, NOTCH signaling dynamically interacts with the tumor immune microenvironment to promote immune suppression and therapy resistance, as described in the following section.

**Table 2:** Therapeutic targeting of NOTCH signaling in GBM.

Therapeutic agents	Type/mechanism	Preclinical evidence	Clinical findings	Limitations	Ref.
MRK003	γ-secretase inhibitor (GSI)	↓ GSC proliferation; ↓ CD133, Nestin, BMI-1, OLIG2; ↑ radiosensitivity in CD44^high/CD133^low GSCs	Not yet in clinical trials for GBM	Effective in targeting resistant GSC populations	(108)
DAPT	GSI	Enhances TMZ/radiation response; ↓ NOTCH targets (e.g., Hes1); synergy with α-secretase inhibitors	Not clinically tested in GBM	Useful in combination therapies to suppress NOTCH and tumor-promoting factors (LIF, YKL-40)	(107, 118)
RO4929097	GSI	↓ GSC proliferation; ↓ spheroid formation; ↑ survival in mouse GBM models (monotherapy + combo)	Phase 0/I Trial (NCT01119599): ↓ NICD, ↓ CD133+ cells; Phase I combo with bevacizumab (NCT01189240): partial responses, tolerability	Auto-induces CYP3A4 → rapid metabolism; NOTCH redundancy	(109, 111, 112, 118)
CB-103	Small molecule NOTCH transcriptional complex inhibitor (downstream of γ-secretase)	Bypasses γ-secretase, directly blocks NOTCH target gene activation	Ongoing Phase I/IIa trial (NCT03422679)	May reduce toxicity and overcome resistance to GSIs	(116)
NOTCH1-specific antibodies	Monoclonal antibodies targeting NOTCH1	↓ tumor growth with limited GI toxicity vs. pan-NOTCH inhibition	Not yet tested clinically in GBM	Isoform-specific targeting reduces systemic side effects	(117)
INCB3619	Dual ADAM10/17 (α-secretase) inhibitor	↓ NOTCH activity (↓ Hes1, Hey1); ↓ GSC growth; ↑ survival in GBM mouse models; synergy with DAPT	Not in clinical trials yet	Targets upstream of γ-secretase; combinatorial potential with GSIs	(118)
Combination therapies (e.g., GSI + TMZ/radiation)	Multi-targeted approach	↓ stemness, ↓ AKT/STAT3, ↓ LIF/YKL-40; ↑ radiosensitivity and chemosensitivity	Clinical trials (e.g., RO4929097 + bevacizumab) show modest benefit	Combination more effective than monotherapy; need better patient stratification	(108, 109, 111, 112, 118)

Clinical trial identifiers: RO4929097 (NCT01119599, NCT01189240), CB-103 (NCT03422679). GSI, γ-secretase inhibitor; NICD, NOTCH intracellular domain; GSC, glioma stem cell; TMZ, temozolomide; DLL3, Delta-like ligand 3, Ab, antibody; LIF, Leukemia Inhibitory Factor; YKL-40/CHI3L1, GBM-associated glycoprotein; ADAM10/17, α-secretases; GI, Gastrointestinal.

## NOTCH as an integrative regulator of the GBM microenvironment

Rather than acting through isolated cell-specific effects, NOTCH signaling in GBM operates across interconnected cellular compartments to establish a coordinated immunosuppressive network. In this framework, tumor-intrinsic NOTCH activation, particularly within GSCs, initiates the production of cytokines, chemokines, and angiogenic factors that reshape the surrounding TME. These signals subsequently drive the recruitment and polarization of myeloid populations, impair antigen presentation, and promote T-cell dysfunction. Thus, the immunosuppressive TME should be viewed not as a collection of independent NOTCH-mediated effects, but as the outcome of a multi-step, hierarchical process linking tumor cell signaling to systemic immune dysregulation. Because these effects are mediated by multiple cellular compartments with distinct biological functions, understanding NOTCH signaling in GBM requires consideration of its cell-type-, receptor-, and ligand-specific activities.

## Cellular context-dependence of NOTCH signaling in GBM

An important consideration in interpreting the role of NOTCH signaling in GBM is its strong dependence on cellular context. Although NOTCH activity is frequently associated with tumor progression, stemness, and therapeutic resistance in glioma cells and GSCs, its effects in immune and stromal compartments may differ substantially. In GSCs, NOTCH1–HES1/HEY1 signaling supports self-renewal, inhibits differentiation, and contributes to resistance to radiation and chemotherapy (70–72). NOTCH signaling also interacts with PI3K/AKT, STAT3, NF-κB, and hypoxia-associated HIF-1α pathways, further supporting GSC maintenance and aggressive tumor phenotypes (73, 78–80). In contrast, within the immune compartment, NOTCH signaling can regulate myeloid-cell states and T-cell differentiation, with outcomes that depend on the cellular and molecular context (19–21, 64). In GBM, NOTCH-associated signaling in TAMs and microglia has been linked to immunoregulatory polarization and production of tumor-supportive mediators, including IL-10, TGF-β, CCL2, and matrix metalloproteinases (21, 124, 125). However, NOTCH activity in T cells cannot simply be considered immunosuppressive, because NOTCH also has established roles in T-cell lineage specification and effector differentiation (19–21, 64). This distinction is particularly relevant when considering systemic effects of tumor-associated NOTCH signaling, as glioma-derived JAG1 can alter thymopoiesis through NOTCH1-dependent mechanisms contributes to changes in peripheral T-cell composition (126–128). NOTCH signaling is similarly heterogeneous in endothelial and stromal compartments. In the GBM vasculature, DLL4 and JAG1 participate in regulating vascular organization and the perivascular niche associated with GSC maintenance (17, 23, 100, 101). However, is should be noted that these ligands are not functionally interchangeable. Differential enrichment of DLL4 and JAG1 has been observed across distinct GBM microvascular patterns, supporting ligand-specific regulation of vascular behavior (23). Thus, the biological consequences of NOTCH signaling depend not only on whether the pathway is activated, but also on which receptor, ligand, and cellular compartment are involved. Collectively, these observations indicate that NOTCH should not be viewed as a uniformly oncogenic or immunosuppressive pathway in GBM. NOTCH1–4 and their ligands can generate distinct, and in some settings, opposing biological effects depending on the responding cell and molecular environment. This context dependence provides an important rationale for selective rather than indiscriminate NOTCH targeting in GBM.

## NOTCH as a multicellular driver of TME remodeling

Failure to treat patients with metastatic or recurrent GBM is largely attributed to tumor heterogeneity and the difficulty of effectively targeting the TME (129). Within this context, NOTCH signaling functions as a central regulator of the TME, exerting immunomodulatory effects through interconnected transcriptional, epigenetic, and intercellular mechanisms. Importantly, NOTCH activity is not restricted to tumor cells but extends to endothelial cells, astrocytes, microglia, and TAMs, forming a dynamic intercellular signaling network (15, 72, 130). Disruption of NOTCH-mediated, dynamic, and interdependent interactions among different components of the TME can drive tumor progression through various mechanisms: *(i)* Transcriptional upregulation of immune checkpoint molecules, like PD-L1 and B7-H4 that inhibit cytotoxic T cell activation and drive T cell exhaustion (131); *(ii)* Suppression of antigen presentation, including the downregulation of major histocompatibility complex (MHC) class I and co-stimulatory molecules, thereby impairing CD8^+^ T cell recognition of tumor antigens and facilitating escape from immune attack (132); and *(iii)* Polarization of myeloid cells, with TAMs adopting M2-like immunosuppressive states and microglia shifting toward immunoregulatory phenotypes, promotes the release of anti-inflammatory cytokines, matrix-remodeling enzymes, and pro-angiogenic factors, creating a tumor-supportive niche (133, 134). These mechanisms generate a tolerogenic immune milieu, provide an immunosuppressive environment, and enhance GBM tumorigenicity, recurrence, and therapy resistance. These interactions are schematically represented in **Figure 3**. Upregulation of NOTCH1, Dll4, and downstream transcriptional targets, such as Hey1 and Hes1, has been consistently observed in patient-derived GBM tissues and has been linked to aggressive tumor behavior and poor clinical outcomes (56, 135, 136). Within the TME, astrocytes and microglia are key TME components that support GBM growth and progression (137). The spatial proximity between GBM cells, glial cells, and immune cells facilitates a direct cell-to-cell contact network, enabling localized Dll4-NOTCH signaling activation, thereby amplifying both pro-angiogenic and immunosuppressive pathways in a subset of GBM tissues (26, 136). This endothelial–glial immune crosstalk plays a dual role for NOTCH signaling: driving tumor cell survival and stemness while modulating immune-vascular niches (138, 139). Targeting this axis may thus disrupt this pathological signaling network and enhance the efficacy of immunotherapeutic strategies in GBM. In a comprehensive review, Li et al. provided evidence that aberrant NOTCH signaling modulates diverse immune cell populations, including TAMs, MDSCs, regulatory T cells, and dendritic cells, often promoting immunosuppressive phenotypes in TME (21). This signaling cascade induces the immunosuppressive condition by enhancing VEGF-mediated angiogenesis and supporting the recruitment and reprogramming of innate immune cells. Microglia and macrophages, which infiltrate gliomas extensively, secrete matrix metalloproteinases as well as cytokines under the influence of NOTCH and NF-κB. This further enhances tumor invasion, ECM remodeling, and aberrant vascularization in GBM (124, 125, 140–142). Therapy-induced modulation of this network is also evident. In oncolytic virotherapy models, viral infection triggers JAG1-mediated NOTCH activation in TAMs, initiating a feed-forward loop involving CCL2-driven recruitment of MDSCs, M2 polarization, and suppression of CD8^+^ T-cell responses. Pharmacological inhibition of NOTCH reverses these effects, highlighting its role in adaptive immune resistance and supporting combinatorial therapeutic strategies (61) (73). Collectively, these findings position NOTCH signaling as a central coordinator of multicellular interactions that drive immunosuppressive remodeling of the GBM microenvironment.

**Figure 3:**
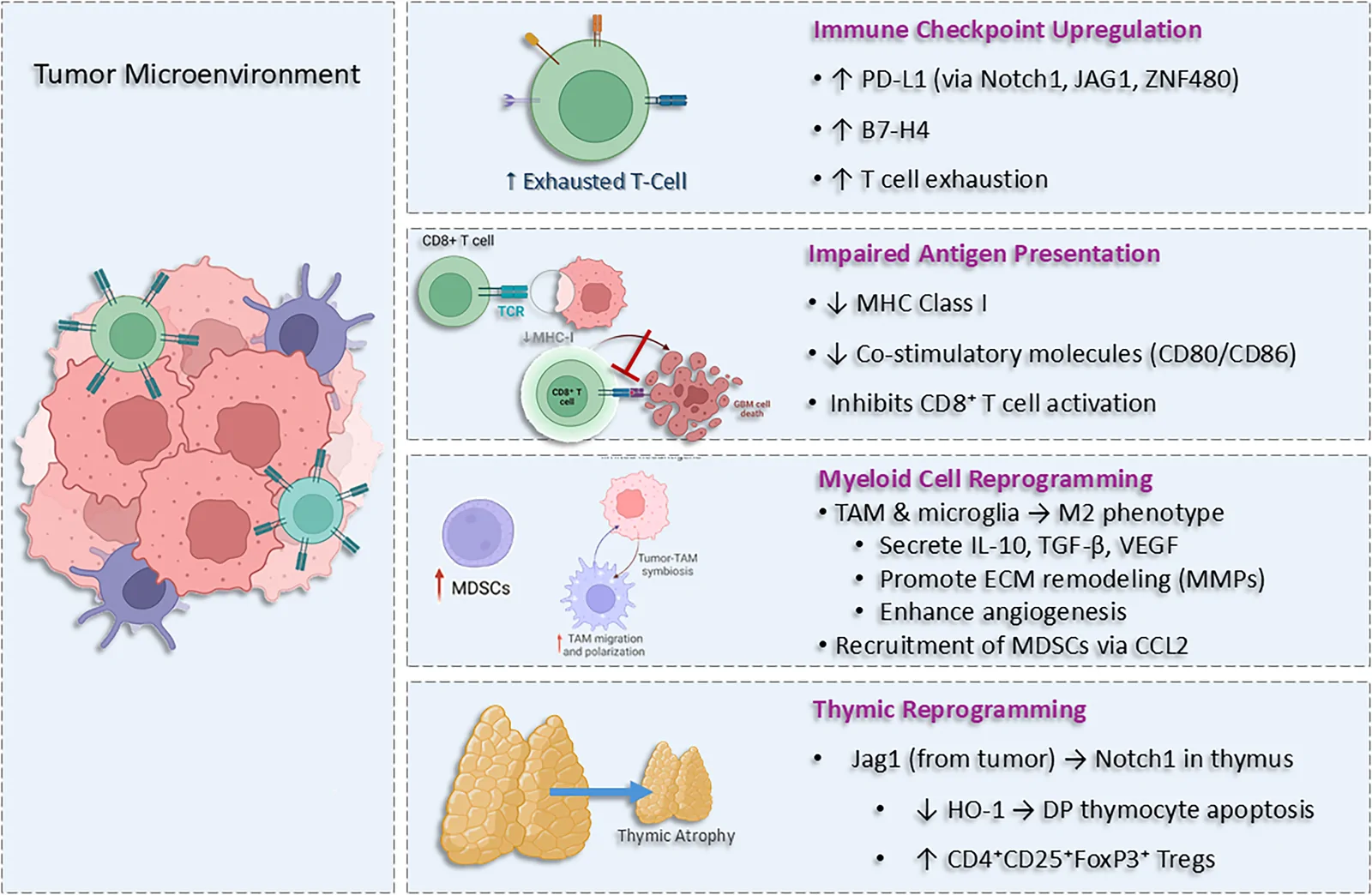
NOTCH signaling-mediated immunosuppression in glioblastoma (GBM). This schematic illustrates how NOTCH signaling modulates both local and systemic immunosuppressive mechanisms in GBM. Locally, NOTCH activation promotes immune checkpoint upregulation (e.g., PD-L1, B7-H4), drives polarization of TAMs and microglia toward an M2-like phenotype, recruits MDSCs and Tregs, and supports the formation of a vascular niche that restricts T-cell infiltration. Systemically, GBM-derived Jagged1 activates thymic NOTCH1 signaling, leading to heme oxygenase-1 (HO-1) downregulation, thymic atrophy, loss of CD4^+^-CD8^+^ thymocytes, and expansion of regulatory T cells, contributing to peripheral lymphopenia and impaired anti-tumor immunity. Note: Abbreviations: GBM, glioblastoma; TME, tumor microenvironment; NICD, NOTCH intracellular domain; TAMs, tumor-associated macrophages; MDSCs, myeloid-derived suppressor cells; Tregs, regulatory T cells; PD-L1, programmed death-ligand 1; B7-H4, B7 homolog 4; HO-1, heme oxygenase 1.

## NOTCH and immune checkpoint regulation

One of the most direct mechanisms by which NOTCH signaling mediates immune evasion in GBM is through the regulation of immune checkpoint molecules that suppress T-cell activation and function. Targeting immune checkpoints in GBM, for example, via HIF-2α inhibition, can remodel the immune infiltrate, shifting the TME from an immunosuppressive condition to a state that supports a stronger and sustained therapeutic response (143). Similarly, NOTCH signaling can shape the immune microenvironment of LGG. In an integrative analysis using TCGA datasets, Shi et al. developed a quantitative NOTCHScore, revealing that high NOTCH activity correlates with poorer prognosis and increased immune checkpoint expression, particularly PD-L1, in LGG (62). These findings indicate that NOTCH signaling contributes to immune evasion and tumor progression across gliomas, regardless of their grade or intrinsic aggressiveness. Among NOTCH pathway components, JAG1 emerged as a critical regulator of LGG cell proliferation and migration. Mechanistically, JAG1 promotes PD-L1 expression via the PI3K–Akt axis and the vitamin D receptor, linking NOTCH activation to immunosuppressive signaling. Knockdown of JAG1 not only impaired tumor cell growth but also disrupted immune checkpoint regulation, reinforcing its functional relevance in glioma immune escape (62). These observations are consistent with broader findings that gliomas exploit NOTCH signaling to modulate the TME, promoting regulatory T cell (Treg) recruitment, TAM polarization, and T-cell dysfunction (8, 9, 144). Given the clinical success of immune checkpoint inhibitors (ICIs) in other solid tumors (145), their application in GBM has been explored (146). Although long-term responses to ICIs in GBM remain below 10% (147); monitoring temporal changes in the tumor-specific genetic profile could serve as a predictive biomarker of therapy response in patients with GBM (148). Furthermore, targeting NOTCH components, such as JAG1, in combination with ICIs or alone has been proposed as a potential immunotherapeutic approach for gliomas, including LGG. Beyond canonical NOTCH ligands and receptors, emerging evidence highlights additional upstream regulators that converge on the NOTCH–immune checkpoint axis to reinforce immunosuppressive signaling in glioma. Zinc finger proteins are key transcriptional regulators in GBM that shape the TME by driving angiogenesis, immune suppression, invasion, and cellular plasticity (149, 150). Recent evidence identifies ZNF480, a member of the Zinc finger protein family, as a novel transcriptional regulator contributing to glioma pathogenesis through NOTCH pathway activation and immune modulation. In LGGs, high ZNF480 expression independently predicts poor prognosis and correlates with NOTCH signaling enrichment, as revealed by gene set enrichment analysis (151). ZNF480 is functionally associated with an immunosuppressive TME, characterized by elevated M2 macrophage infiltration and PD-L1 upregulation, aligning with NOTCH-mediated immune evasion mechanisms. Experimental silencing of ZNF480 reduces its oncogenic expression profile, highlighting its potential as a therapeutic target. These findings position ZNF480 at the intersection of NOTCH-driven stemness and immune suppression, offering a dual-target strategy to overcome therapy resistance in LGG (151). It has been suggested that NUP205, a core nucleoporin of the nuclear pore complex (NPC), is a potential contributor to glioma progression and immune evasion through NOTCH pathway regulation. In a comprehensive transcriptomic analysis of LGG, Liang et al. reported that NUP205 is significantly overexpressed in aggressive LGG subtypes (IDH-wildtype, 1p/19q non-codeleted) and correlates with worse prognosis and an immunosuppressive TME (152). NUP205 modulates both NOTCH signaling and cell cycle regulators, implicating NPC dysfunction in the transcriptional control of oncogenic and immune-related pathways. High NUP205 expression was linked to enhanced PD-L1 expression and elevated infiltration of M2-like macrophages, suggesting that NUP205 may facilitate immune escape through the enhancement of NOTCH-mediated immune checkpoint activation and myeloid reprogramming (152). However, it is critical to note that these immunoregulatory links are currently supported primarily by in silico correlations and expression profiling. These findings are consistent with prior studies demonstrating that NOTCH activation via JAG1 or NOTCH1 promotes PD-L1 upregulation and suppresses T-cell function in glioma. As such, NUP205 represents a novel upstream node in the NOTCH–PD-L1 axis and may serve as a dual-purpose therapeutic target, simultaneously attenuating glioma growth and reversing immune evasion. Together, these findings identify ZNF480 and NUP205 as regulatory nodes that connect transcriptional control with immune checkpoint activation and myeloid reprogramming in glioma. Collectively, these findings indicate that NOTCH signaling promotes immune evasion by directly regulating checkpoint molecule expression, thereby limiting effective T-cell–mediated anti-tumor responses.

In addition to PD-1/PD-L1 signaling, increasing evidence indicates that multiple inhibitory checkpoints contribute to the complex and redundant immune-suppressive network of GBM. Among these, TIGIT, LAG-3, and TIM-3 are inhibitory immune checkpoints expressed on both lymphoid and myeloid cells that can suppress antitumor immune responses and contribute to resistance to single-checkpoint blockade. An investigation has identified an unexpected role for TIGIT in GBM-associated myeloid cells. Asad et al. demonstrated broad TIGIT expression in myeloid populations within human GBM and showed that GBM-derived extracellular vesicles can induce TIGIT expression in monocytes. TIGIT knockdown reduced the immunosuppressive polarization of these cells and restored T-cell function in an NLRP3-dependent manner, with TIGIT and NLRP3 showing substantial co-expression. These findings suggest that TIGIT functions not simply as a marker of T-cell exhaustion but as a regulatory node controlling the balance between immunosuppressive myeloid activity and NLRP3-associated pro-inflammatory responses in the TME of GBM (153). This myeloid checkpoint axis is particularly relevant to therapeutic strategies aimed at reprogramming the immunosuppressive TME. LAG-3 (CD223) represents another layer of immune suppression, predominantly through exhausted T cells and regulatory T cells. In human glioma, LAG-3^+^ tumor-infiltrating lymphocytes are enriched in IDH-wildtype GBM and are associated with CD8^+^ T-cell infiltration, PD-1 expression, and PD-L1-positive tumor cells, supporting its relationship with an already activated but functionally suppressed immune microenvironment (154). Mechanistically, LAG-3 can engage several ligands, including MHC-II, FGL1, and galectin-3, and its functional cooperation with PD-1 can reinforce T-cell dysfunction (155–157). Of particular relevance to GBM, a recent study demonstrated that radiotherapy induces galectin-3 expression in GSCs through EGR1, thereby activating the Gal3–LAG-3 axis and promoting exhaustion of both CD4+ and CD8+ T cells in recurrent GBM. The combination of EGR1 inhibition and LAG-3 blockade suppressed tumor growth in orthotopic models, pointing to a therapy-induced mechanism of immune escape (158).

TIM-3 (HAVCR2) provides an additional checkpoint linking adaptive immune exhaustion to myeloid and innate immune regulation. TIM-3 is expressed by T cells, NK cells, macrophages, and dendritic cells, as well as in GBM tumor cells. Its interaction with ligands, such as galectin-9 and CEACAM1, can suppress T-cell and NK-cell activity while promoting immunosuppressive myeloid phenotypes (159, 160). In GBM, TIM-3 expression has been associated with higher tumor grade, an IDH-wildtype/mesenchymal phenotype, M2-like macrophage accumulation, and tumor-promoting signaling through a Gal-9/TIM-3–IL-6/NF-κB axis (161, 162). TIM-3 may also suppress innate immune sensing by limiting HMGB1-mediated uptake of tumor-derived nucleic acids and cGAS–STING activation in dendritic cells, thereby further weakening antitumor immunity (163, 164).

Collectively, these findings support a model in which GBM immune escape is maintained by interconnected rather than independent checkpoint pathways. Consistent with this concept, a recent clinical study of recurrent GBM showed that nivolumab treatment activated intratumoral T cells but simultaneously induced compensatory upregulation of TIGIT, LAG-3, TIM-3, and CTLA-4, including increased populations co-expressing multiple checkpoints. Thus, NOTCH-driven immune suppression may be viewed within a broader network in which developmental signaling, myeloid reprogramming, T-cell exhaustion, and innate immune regulation converge to produce a highly resilient immunosuppressive TME. Therapeutically, these observations provide a rationale for multi-target or context-dependent checkpoint strategies, instead of inhibition of a single checkpoint.

## Crosstalk with developmental pathways (Wnt and Hedgehog)

In addition to its direct immunoregulatory functions, NOTCH signaling operates within a broader developmental signaling network that amplifies tumor progression and immune suppression. Crosstalk with pathways such as Wnt and Hedgehog reinforces NOTCH-driven transcriptional programs and stabilizes immunosuppressive phenotypes. Pan-cancer analyses have identified WNT5A as a key immunoregulatory hub that co-activates NOTCH and Hedgehog pathways to facilitate tumor progression (165). The interaction between Wnt and NOTCH signaling pathways controls development and the pathogenesis of various diseases (166). WNT5A, a non-canonical Wnt ligand, is upregulated in GBM and promotes cell infiltration in TME (167). NOTCH signaling is active in spheroid-forming GSCs, and inhibition of NOTCH signaling suppresses the expression of Ror1, a receptor or co‐receptor for Wnt5a, in GBM cells (168, 169). WNT5A is consistently overexpressed and correlates with poor prognosis, increased immune checkpoint expression (such as programmed death-ligand 1 (PD-1) and CTLA-4), and enrichment of NOTCH transcriptional programs in gliomas. WNT5A amplifies NOTCH-driven immune suppression by promoting Treg recruitment, M2 macrophage polarization, and co-regulating pro-inflammatory cytokines, such as IL-12, IL-23, and TNF-α through NOTCH1 (168).

Interestingly, tumors with high WNT5A expression exhibit features of “immune-inflamed” (hot) tumors, characterized by increased T-cell infiltration, elevated interferon-γ signaling, enhanced PD-1 expression, and high tumor mutational burden, which are generally predictive of better responses to ICIs (170). However, WNT5A-driven gliomas often display an “immune-hot but suppressed” TP1 subtype, where despite abundant immune infiltration, clinical outcomes remain poor, supporting the role of WNT5A as a co-driver of NOTCH-mediated immune evasion (168). Moreover, dysregulation of the Wnt/planar cell polarity (PCP) pathway contributes further to GBM aggressiveness. VANGL2, a core PCP gene, has been linked to genes enriched in NOTCH signaling and stem cell pluripotency pathways. Functional studies have demonstrated that VANGL2 enhances glioma cell migration, invasion, proliferation, and tumor sphere formation, highlighting its role as a mediator of glioma progression (171). Thus, WNT5A represents both a functional modulator and a potential biomarker of NOTCH activity, with therapeutic value when co-targeted alongside GSIs or ICIs to disrupt this immunosuppressive circuit. These findings demonstrate that NOTCH signaling functions within an interconnected signaling network that integrates developmental and immune pathways to sustain immunosuppression. Importantly, the evidence supporting these NOTCH pathway interactions is heterogeneous. Among the currently described crosstalk mechanisms, the NOTCH–Hedgehog interaction provides a particularly informative example of functional pathway compensation, as increased Hedgehog activity following NOTCH inhibition and direct interaction between HES1 and GLI1 have been demonstrated in GBM neurosphere and primary GBM cells (172). Consistent with this mechanism, combined inhibition of NOTCH and Hedgehog signaling produces greater effects on apoptosis, cell growth, and colony formation than either treatment alone (172). In contrast, several Wnt–NOTCH associations, including those involving WNT5A, are supported primarily by transcriptomic, observational, and preclinical studies and should therefore be considered hypothesis-generating rather than definitive evidence of clinical efficacy. These observations nevertheless provide a rationale for investigating pathway-selective combination strategies and for exploring molecular features of pathway co-activation as potential biomarkers for patient stratification. Such approaches remain preclinical and require validation before clinical application.

## Systemic NOTCH-mediated immunosuppression: thymic atrophy and Treg bias in glioma

While NOTCH signaling has been extensively characterized within the local TME, emerging evidence indicates that its immunoregulatory effects extend beyond the tumor site to influence systemic immune homeostasis. In particular, NOTCH plays a central role in T-cell development, where receptor–ligand interactions within distinct thymic compartments govern lineage commitment, maturation, and functional polarization (173). NOTCH signaling regulates the antitumor immune response, and higher numbers of T cells (particularly activated CD8+ cytotoxic T lymphocytes and Th1 cells) are associated with improved survival in various malignancies (174). In the context of glioma, tumor-derived signals can disrupt this tightly regulated process. El Andaloussi et al. demonstrated that intracranial glioma progression induces profound thymic atrophy through activation of the JAG1–NOTCH1 axis, leading to depletion of CD4^+^CD8^+^ double-positive thymocytes and impaired maturation of single-positive T-cell subsets. Mechanistically, aberrant NOTCH activation suppresses HO-1, a key anti-apoptotic regulator, thereby promoting selective apoptosis of developing thymocytes. This shift in thymic dynamics favors the expansion of CD4^+^CD25^+^Foxp3^+^ regulatory T cells (Tregs), resulting in a systemic immunosuppressive phenotype (126–128). Importantly, bone marrow transplantation experiments confirmed that glioma-derived JAG1 is sufficient to induce these effects, pointing to a tumor-intrinsic mechanism for shaping the peripheral T-cell repertoire. These findings suggest the JAG1–NOTCH1–HO-1 axis as a potentially significant pathway contributing to immune dysregulation in specific preclinical models of glioma. However, further validation in representative models and patient-derived systems is required to determine its broader clinical relevance and status as a primary driver of systemic immunosuppression.

Functionally, these systemic effects are not independent of the TME but instead reinforce local immunosuppressive circuits. The expansion of Tregs and reduction in effective cytotoxic T-cell output from the thymus limit the availability of anti-tumor effector cells, thereby amplifying NOTCH-driven immune evasion within the TME. This bidirectional relationship suggests that glioma leverages NOTCH signaling to coordinate immune suppression across both central and peripheral compartments. Clinically, such systemic immune alterations may contribute to lymphopenia observed in GBM patients and help explain the limited efficacy of immunotherapies that depend on robust T-cell priming. Therapeutically, these findings highlight the need for strategies that selectively modulate NOTCH signaling while preserving essential immune functions, potentially through combinatorial approaches that prevent both local and systemic immunosuppression. Taken together, these findings indicate that NOTCH signaling extends beyond the TME to systemically reshape immune cell development and composition. Importantly, these systemic alterations are not independent of local tumor processes; rather, they reinforce and sustain the immunosuppressive circuitry established within the glioma microenvironment. This bidirectional interaction suggests that NOTCH signaling operates across multiple biological scales, integrating tumor-intrinsic programs with both local and systemic immune regulation.

## Contextual complexity of NOTCH signaling

The complexity introduced by pathway crosstalk helps explain the context-dependent characteristics of NOTCH signaling in glioma. While NOTCH activation is predominantly associated with immune suppression, its functional impact varies depending on cellular context and microenvironmental conditions. Experimental evidence indicates that NOTCH inhibition can impair anti-tumor immunity. In murine glioma models, loss of NOTCH activity reduces MHC class I expression and cytokine production, leading to diminished CD8^+^ T-cell recruitment and enhanced immunosuppressive myeloid activation. Furthermore, NOTCH-deficient tumors exhibit resistance to interferon-γ signaling and macrophage-reprogramming therapies, highlighting a role for NOTCH in maintaining immune competence (165). Transcriptomic analyses support these findings, showing that low NOTCH activity is associated with downregulated interferon responses and activation of oncogenic pathways. These findings indicate that excessive NOTCH signaling promotes immune evasion, while complete suppression may disrupt essential immune surveillance mechanisms (165). Taken together, these data point to the context-dependent nature of NOTCH pathway, where both activation and inhibition can differentially shape immune responses and influence therapeutic outcomes.

## NOTCH as a multi-level immunoregulatory hub

Collectively, the evidence presented positions NOTCH signaling as a multi-level immunoregulatory hub that integrates tumor-intrinsic programs with microenvironmental and systemic immune dynamics in glioma. Rather than acting through isolated mechanisms, NOTCH coordinates a hierarchy of processes spanning tumor cells, immune populations, and stromal components. At the tumor-intrinsic level, NOTCH activation promotes stemness, metabolic adaptation, and the production of immunomodulatory factors. These signals extend into the microenvironment, where NOTCH drives immune checkpoint expression, suppresses antigen presentation, and orchestrates the recruitment and polarization of myeloid cells toward immunosuppressive phenotypes. Beyond these local effects, NOTCH signaling is further reinforced through crosstalk with developmental pathways such as Wnt and Hedgehog, which amplify transcriptional programs associated with immune evasion and tumor progression. This networked signaling architecture enables GBM cells to sustain a stable yet adaptable immunosuppressive niche, even under therapeutic pressure. At the same time, emerging evidence indicates that NOTCH activity contributes to systemic immune regulation, suggesting that its effects extend beyond the tumor site to influence broader immune competence. Importantly, the function of NOTCH signaling is highly context-dependent. While sustained activation predominantly reinforces immune suppression, its complete inhibition may disrupt interferon signaling, impair antigen presentation, and weaken effective T-cell responses. This duality highlights NOTCH not simply as an oncogenic pathway, but as a dynamic regulatory system that balances immune activation and suppression depending on cellular and environmental conditions. The diverse immunosuppressive roles of NOTCH signaling in the glioma TME are summarized in **Table 3**, showing its interactions with tumor, immune, and stromal components that modulate immune escape and therapy resistance. Taken together, these observations support a model in which NOTCH signaling serves as an integrative hub linking tumor plasticity, immune evasion, and therapeutic resistance. This conceptual framework has important implications for treatment strategies, suggesting that selective modulation rather than complete inhibition of NOTCH signaling may be required to effectively restore anti-tumor immunity in glioma. Importantly, the integrative role of NOTCH signaling across tumor, immune, and stromal compartments converges on a central cellular determinant, GSCs. As such, GSCs function not only as a reservoir of tumor plasticity but also as an active signaling hub that links NOTCH-driven stemness programs with coordinated immune suppression, as detailed in the following section.

**Table 3:** Immunosuppressive roles of NOTCH signaling in the glioma TME and systemic immunity.

Mechanism	Key findings	Molecular/cellular players	Functional consequences	Clinical/therapeutic implications	Ref
Local immunosuppression in TME					
Immune Checkpoint Upregulation	NOTCH induces PD-L1, B7-H4 via JAG1-PI3K-Akt/VDR.	NOTCH1, Dll4, JAG1, Hes1/Hey1	T-cell exhaustion, impaired cytotoxicity	Combined NOTCH + ICI therapy may overcome resistance	(56, 62, 135, 136)
Antigen Presentation Suppression	Downregulates MHC-I and co-stimulatory molecules.	NOTCH inactivation (NOTCH1/2 loss)	Reduced CD8^+^ T-cell recognition	NOTCH modulation (not broad inhibition) required	(165)
Myeloid Polarization (TAMs/Microglia)	Drives M2-like phenotypes via Jag1/NF-κB.	TAMs, microglia, CCL2, MMPs	IL-10/TGF-β secretion, angiogenesis, ECM remodeling	GSIs reverse polarization; enhance immunotherapy	(21, 124, 125)
Endothelial–Glial Crosstalk	Dll4-NOTCH promotes vascular-immune niche.	Dll4, VEGF, HIF-1α	Stemness, immune evasion, angiogenesis	Anti-Dll4 therapies disrupt niche	(72, 130)
Treg/MDSC Recruitment	NOTCH activates CCL2/JAG1, recruiting immunosuppressive cells.	JAG1, WNT5A, CCL2	T-cell suppression, tumor progression	JAG1 inhibition + ICIs may improve responses	(61, 165)
Systemic immune dysregulation					
Thymic Atrophy & Altered T-cell Repertoire	Glioma-derived Jag1 disrupts thymopoiesis via NOTCH1.	Jag1, NOTCH1, HO-1	Depletion of DP thymocytes, Treg expansion	Preserving HO-1 may mitigate lymphopenia	(126–128)
Peripheral Lymphopenia	Jag1-NOTCH1 axis reduces naïve T-cell output.	CD4^+^CD8^+^ DP thymocytes, CD4^+^CD25^+^Foxp3^+^ Tregs	Impaired T-cell priming, systemic immunosuppression	Bone marrow-targeted NOTCH modulation	(128)
Therapy resistance mechanisms					
Oncolytic Virotherapy Escape	oHSV induces Jag1 in TAMs, recruiting MDSCs.	Jag1, CCL2, MDSCs	Limits oHSV efficacy	GSIs + oHSV combination therapy	(61)
WNT5A-NOTCH Crosstalk	Co-activates NOTCH to drive Treg/M2 polarization.	WNT5A, IL-12/IL-23, TNF-α	“Immune-hot but suppressed” phenotype	Dual WNT5A + NOTCH blockade	(165)
NUP205-Mediated Immune Evasion	Enhances PD-L1 and M2 infiltration via NOTCH.	NUP205, PD-L1, NOTCH1	Immune checkpoint activation	Target NPC-NOTCH axis	(152)

MHC-I, major histocompatibility complex class I; NF-κB, nuclear factor kappa-light-chain-enhancer of activated B cells; VEGF, vascular endothelial growth factor; HIF-1α, hypoxia-inducible factor 1-alpha; IL-10, interleukin-10; TGF-β, transforming growth factor-beta; CCL2, chemokine (C-C motif) ligand 2; oHSV, oncolytic herpes simplex virus; ICI, immune checkpoint inhibitor; TAMs, tumor-associated macrophages; MDSCs, myeloid-derived suppressor cells; Tregs, regulatory T cells.

## NOTCH signaling in coordinating GSC maintenance and immunosuppression

### NOTCH as a driver of GSC stemness and plasticity

GSCs provide an important cellular interface between NOTCH signaling and the immune microenvironment. Rather than functioning solely as a self-renewing tumor cell population, GSCs can actively participate in remodeling the surrounding niche through cytokine, chemokine, and intercellular signaling. In this context, NOTCH signaling may connect GSC-associated programs with immune regulation and therapeutic adaptation (12). Targeting NOTCHless homolog 1 (NLE1), a negative regulator of NOTCH activity, impairs NOTCH signaling, modulates stem cell marker expression, and sensitizes GSCs to radiation-induced cell death (175). Inhibition of NOTCH signaling appears to reduce the growth and self-renewal capacity of CD133^+^ GSCs both *in vitro* and *in vivo*, indicating its potential role in depleting the stem-like tumor cell reservoir (12). According to Tang et al., GSCs mediate tumor progression, immune evasion, and resistance to therapy, with NOTCH signaling acting as a regulatory pathway linking these processes (176). In the TME, NOTCH cooperates with other developmental pathways, like Wnt/β-catenin and Hedgehog, allowing GSCs to thrive under hypoxic conditions (177). These interactions activate transcriptional programs that maintain stem-like identity and promote phenotypic plasticity and metabolic adaptation, both of which contribute to tumor progression and recurrence (177, 178). In sum, these data suggest NOTCH signaling as a central modulator of GSC plasticity and persistence, forming the foundation for downstream immune modulation.

### NOTCH-driven GSC–immune crosstalk

Beyond maintaining stemness, NOTCH signaling enables GSCs to actively remodel the immune microenvironment through cytokine-mediated communication and immune cell reprogramming. Rather than functioning as passive responders, GSCs leverage NOTCH activation to restructure the immune milieu through the secretion of immunomodulatory factors and engagement of signaling cascades. For instance, interferon regulatory factor 7 (IRF7) amplifies NOTCH output by inducing IL-6, CXCL1, and CCL2, thereby promoting angiogenesis, recruiting TAMs, and activating the JAK/STAT3–Jagged1–NOTCH signaling axis (179). This feed-forward loop reinforces both the GSC phenotype and the establishment of an immune-tolerant TME. Importantly, silencing IRF7 attenuates stemness and immune suppression simultaneously, highlighting its role as a convergent regulatory node (179). These observations demonstrate that NOTCH signaling functions as a bidirectional interface through which GSCs both shape and are reinforced by the immunosuppressive microenvironment.

## Hypoxia–NOTCH–Hedgehog axis in immune evasion

Under hypoxic conditions, NOTCH signaling cooperates with developmental pathways to couple stemness maintenance with immune evasion. Chang and Lai demonstrated that dual activation of NOTCH and Hedgehog signaling defines an aggressive GSC phenotype characterized by elevated stemness markers (EZH2, SUZ12, REST) and localization within hypoxic niches (180). Mechanistically, hypoxia stabilizes HIF-1α, which enhances NOTCH–Hedgehog signaling interactions, thereby sustaining cancer stem cell maintenance. This signaling axis promotes immune escape by increasing Treg infiltration, suppressing MHC class I expression, and reinforcing VEGF-driven angiogenesis. The resulting feedback loop supports tumor survival under stress while limiting immune recognition and therapeutic responsiveness. Together, these findings identify hypoxia-driven NOTCH crosstalk as a key mechanism linking metabolic adaptation to immune suppression in GSCs.

## Transcriptional modulators of NOTCH in GSCs

Fine-tuning of NOTCH signaling in GSCs is mediated by transcriptional regulators that integrate developmental and immune signaling programs. Among these, HES6 emerges as a non-canonical NOTCH effector that modulates transcriptional output and balances glioma stemness with immune regulation. Unlike HES1, HES6 suppresses HES1/ASCL1 activity while enhancing DLL1 expression, thereby reshaping NOTCH signaling dynamics (181). Notably, HES6 is enriched in both CD8^+^ T cells and TAMs and correlates with gene signatures associated with epithelial–mesenchymal transition, Wnt/KRAS signaling, and both pro-inflammatory and immunosuppressive pathways (182). These dual associations suggest that HES6 integrates tumor-intrinsic and immune-related signals within the TME. Overall, transcriptional regulators such as HES6 refine NOTCH activity to coordinate stemness programs with immune modulation.

## Therapeutic targeting of the NOTCH–GSC axis

Targeting NOTCH signaling in GSCs represents a strategy to simultaneously disrupt tumor maintenance and restore immune surveillance. Evidence indicates that Hedgehog signaling is increased following NOTCH pathway inhibition, and a direct interaction between Hes1, a transcriptional repressor, and Gli1, a major downstream effector of Hedgehog signaling, has been observed in both GBM neurosphere lines and primary GBM cells. Simultaneous targeting of both the NOTCH and Hedgehog pathways significantly enhances apoptosis and more effectively inhibits cell growth and colony formation compared to monotherapy (183). These findings revealed that targeting NOTCH either directly or in combination with Hedgehog inhibitors, hypoxia modulators, and/or checkpoint blockade therapies offers a potential strategy to simultaneously disrupt GSC maintenance and restore immune surveillance within the glioma TME (184). Together, these approaches underscore the therapeutic potential of targeting the NOTCH–GSC axis within a broader network context.

## Primary cilia as a platform for NOTCH signaling integration

Subcellular signaling platforms such as primary cilia further contribute to the regulation of NOTCH-dependent pathways in GSCs. Primary cilia are microtubule-based organelles that mediate cellular signaling by both receiving and integrating extracellular cues within the TME, thereby contributing to oncogenesis across multiple cancer types (185). They contribute significantly to regulating neural stem cell proliferation and migration, hosting important pathways such as NOTCH, Hedgehog, and WNT (186). In GBM, the impact of primary cilia may depend on the abundance of ciliated tumor cells and their signaling functions, with evidence suggesting that glioma cilia can either restrict or promote gliomagenesis (187). By integrating extracellular signals, primary cilia fine-tune NOTCH pathway activity and coordinate NOTCH-driven communication between tumor cells and the surrounding microenvironment, thereby linking microenvironmental sensing to stem cell adaptation and therapeutic resilience. These observations suggest that subcellular signaling architecture adds a regulatory layer to NOTCH-mediated control of GSC behavior and immune interactions. As detailed in the following section, this regulatory framework enables GSCs to exploit NOTCH signaling to survive genotoxic stress and maintain an immunosuppressive niche that ultimately impairs therapeutic efficacy.

## The NOTCH–GSC–immune axis in glioma therapy resistance

Therapeutic resistance in GBM reflects the interaction between resilient tumor cell populations and a protective TME. Evidence suggests that the NOTCH signaling pathway contributes to this interaction by connecting GSC-associated survival programs with immune and stromal mechanisms (177, 188). Specifically, the activation of NOTCH1 sustains GSC persistence through downstream PI3K/AKT/mTOR pathway stimulation, enabling tumor repopulation following radiotherapy and correlating with poor clinical outcomes (189). Beyond contributing to intrinsic resistance, NOTCH signaling plays a role in bridging therapy resistance with immune evasion. Zhang et al. reported that JAG1 mediates radiotherapy resistance and is downregulated when radiotherapy is combined with colony-stimulating factor 1 (CSF-1) blockade, which depletes immunosuppressive TAMs (190). Moreover, high expression of JAG1, NOTCH1, and DLL1 correlates with poor prognosis in glioma patients undergoing radiotherapy (62). This resistance is reinforced by YAP1, a Hippo pathway effector, that transcriptionally activates JAG1, forming a YAP1–JAG1–NOTCH1 loop that sustains both tumor progression and immune suppression (191).

In a complementary model, Chockalingam et al. showed that overexpression of macrophage colony-stimulating factor in glioma cells promotes the recruitment of TAMs while simultaneously inducing resistance to 5-fluorouracil via NOTCH1-mediated EMT and expansion of a CD24high/CD44low CSC population (130, 192). These findings point to a dual mechanism whereby TAM-derived immunosuppressive cytokines enhance NOTCH activation, which in turn promotes chemoresistant CSC expansion, creating a mutually reinforcing feedback loop. Transcriptomic analyses further reveal that GSCs display heightened NOTCH dependency, contributing to resistance against TMZ and radiotherapy (109, 193, 194). Although NOTCH signaling is widely dysregulated in gliomas, clinical translation of NOTCH inhibitors remains challenging (193). For instance, RO4929097, a γ-secretase inhibitor, showed limited efficacy as a monotherapy in glioma models, despite robust *in vitro* activity and frequent NOTCH pathway activation (195). Its benefit emerged only when combined with genotoxic agents like TMZ or irradiation, highlighting the context-specificity of NOTCH dependency (195). Furthermore, MDSCs use complex immunosuppressive strategies adapted to the TME, making their interaction with GSCs a key target for therapy (102). Notably, treatment response correlated inversely with IL-6 expression, linking NOTCH signaling to IL-6-mediated MDSC recruitment and TAM polarization. Failure of RO4929097–mTOR combination therapy further underscores the resilience of the immunosuppressive niche *in vivo*. Collectively, the data support NOTCH signaling as a molecular integrator of CSC-driven resistance and immune escape, and suggest that effective treatment will require combinatorial strategies targeting NOTCH alongside immune-modulatory agents, such as checkpoint inhibitors, CSF-1R blockers, or TAM reprogramming therapies (196). The MEOX2-NOTCH axis refers to the interaction between the transcription factor MEOX2 (mesenchyme homeobox 2) and the NOTCH signaling pathway, which together influence self-renewal and stemness maintenance of GSCs. Targeting this axis facilitates tumor invasion, metastatic progression, and immune evasion via the enhancement of inflammatory and proteolytic signaling, presumably via the modulation of the NF-κB signaling pathway (197). These data collectively position Notch signaling as a molecular integrator of CSC-driven resistance and immune escape, and suggest that effective treatment will require combinatorial strategies targeting Notch alongside immune-modulatory agents, such as checkpoint inhibitors, CSF-1R blockers, or TAM reprogramming therapies.

## MicroRNA regulation of NOTCH-driven GBM stemness and immune evasion

Directly targeting NOTCH signaling has proven effective in diminishing therapy-resistant GSC populations and disrupting reprogramming of the immunosuppressive TME; however, the effects of modulating the upstream regulatory networks that control NOTCH activity are still under investigation. Among these regulators, microRNAs (miRNAs) have emerged as critical modulators of NOTCH-driven phenotypes in glioma (198). These small non-coding RNAs can act as tumor suppressors or oncogenic miRNAs (oncomiRs), influencing key processes in GBM pathology, such as GSC maintenance, immune evasion, and therapy resistance (199). In this part, we addressed the complex interplay between miRNAs and NOTCH signaling in GBM, highlighting their dual role as biomarkers and therapeutic targets in overcoming treatment resistance.

In GBM, tumor-suppressive miRNAs, like the miR-34 family (miR-34a, miR-34c-3p, miR-34c-5p), are frequently downregulated. Their expression inversely correlates with NOTCH1 and NOTCH2 activity and glioma invasiveness. Restoration of miR-34 or miR-31 levels induces apoptosis, cell cycle arrest, and loss of stem-like traits, effectively disrupting NOTCH-driven oncogenic programs (200, 201). On the other hand, glioma-derived oncomiRs, such as miR-92a, are secreted to suppress natural killer cell-mediated immunity by downregulating the expression of key immune molecules involved in killing target cells and orchestrating immune responses, such as interferon-γ (IFN-γ), perforin, and Fas ligand (202). Khwaja et al. identified miR-146b, an immune-modulatory microRNA, as significantly upregulated in post-therapy recurrent GBM and markedly associated with the activation of NOTCH, Wnt, and VEGF signaling pathways (203). miR-146b disrupts anti-tumor immune responses by influencing NF-κB signaling in myeloid cells, which can enhance the NOTCH-mediated recruitment of immunosuppressive TAMs and contribute to an immune-evasive TME (136, 203). Furthermore, the co-activation of NOTCH signaling and miR-146b in recurrent GBM supports GSC populations by promoting NOTCH-dependent self-renewal and intrinsic resistance to chemo-radiation (203, 204). Therapy-induced stresses (Such as hypoxia) upregulate miR-146b and reactivate NOTCH, together promoting an immunosuppressive TME that drives tumor recurrence. Targeting this axis, for example, by combining NOTCH inhibitors and miR-146b antagonists could disrupt the feedback loop between glioma stemness, immune evasion, and therapeutic resistance (203). Furthermore, downregulation of miR-146b-5p enhances glioma cell proliferation, inhibits apoptosis, and is associated with unfavorable survival outcomes. Acting as a tumor suppressor, miR-146b-5p mediates TMZ resistance in GBM through negative regulation of tumor necrosis factor receptor-associated factor 6. miR-146b-5p overexpression restores TMZ sensitivity in resistant GBM cells (205).

Similarly, miR-33a acts as an oncogene in glioma by downregulating its target SIRT6, which leads to reduced apoptosis through activation of the JAK2/STAT3 pathway, promoting glioma cell survival (206). miR-33a has been defined as a master regulator of GSC self-renewal through concurrent activation of the NOTCH and PKA pathways. Elevated miR-33a expression correlated with poor prognosis in GBM patients, and its knockdown suppressed GSC tumorigenicity, while its overexpression conferred stem-like properties to non-GICs. miR-33a overexpression downregulates phosphodiesterase 8A and a UV radiation resistance-associated gene, while upregulating their downstream effectors p-CREB and the NOTCH intracellular domain in glioma cells (207). It is important to note that the expression of various miRNAs varies among different GBM tissue samples. miR-148a is frequently upregulated in GBM and shows a strong correlation with genes involved in hypoxia response and extracellular matrix remodeling. In contrast, elevated values of miR-31 are observed in a small subset of GBM and are associated with genes related to cell proliferation and immune responses (201). Dual pharmacological inhibition of NOTCH by DAPT and PKA by KT5720 synergistically impaired GSC growth, whereas combined activation of both pathways (via Forskolin and JAG1) induced stemness in non-GICs (207). These findings position miR-33a as a central node linking NOTCH-dependent self-renewal and PKA-mediated proliferation, offering a compelling therapeutic target. Given that both NOTCH and PKA pathways contribute to PD-L1 upregulation and T-cell exclusion, targeting miR-33a or its downstream effectors may offer dual therapeutic advantages, disrupting GSC self-renewal and reprogramming the immune microenvironment. Taken together, miRNA dysregulation, particularly miR-34, miR-146b, and miR-33a, contributes to NOTCH hyperactivation and immunosuppressive rewiring in GBM. These interactions enable glioma recurrence and resistance to therapy. Targeted modulation of the miRNA–NOTCH axis provides a novel therapeutic approach, particularly in recurrent or immune-resistant GBM.

## ECM-driven modulation of NOTCH signaling in glioma immunosuppression and therapy resistance

ECM is no longer viewed as a passive structural scaffold but rather as a dynamic and bioactive component of TEM that contributes to immune modulation, tumor progression, and therapeutic resistance (208). Recent evidence has highlighted an ECM–NOTCH–immune regulatory axis, in which dynamic changes in matrix composition reciprocally affect both tumor cell behavior and immune cell function (209). CD47 is a cell surface protein that acts as an immune regulator by providing a “don’t eat me” signal and thereby inhibiting the phagocytosis of healthy cells (210). CD47 loss-of-function enhances phagocytosis while paradoxically activating NOTCH signaling. This activation induces tenascin-C (TNC) upregulation, an extracellular matrix glycoprotein overexpressed in GBM and correlated with poor clinical outcomes (211). The NOTCH-mediated TNC secretion drives M2-like TAM polarization, promotes ECM remodeling, impairs T cell infiltration, and exacerbates immunosuppression. Furthermore, autocrine TNC-NOTCH signaling loops in GSCs reinforce stemness properties and therapy resistance (76). These observations imply that CD47-targeted monotherapies may trigger compensatory activation of NOTCH signaling, pointing to the potential of combinatorial approaches, such as combining CD47 blockade with γ-secretase inhibitors, to prevent adaptive immune evasion in GBM (211). In a complementary study, Li et al. characterized ZR30, an engineered fibulin-3 (EFEMP1) derivative that possesses both ECM–modulating and NOTCH-inhibitory properties. Using orthotopic GBM models, evidence indicates that ZR30 effectively targets the EGFR/NOTCH/AKT signaling axis, a central hub of therapeutic resistance, while disrupting critical tumor-ECM interactions (212). At the molecular level, ZR30 suppressed MMP2 activity, a NOTCH-dependent metalloproteinase pivotal for invasion, ECM degradation, and immune exclusion, and modulated downstream effectors by downregulating the proliferative transcription factor FOXM1 while restoring expression of the anti-invasive semaphorin SEMA3B (194, 212). These coordinated actions support the capacity of ZR30 to simultaneously address tumor heterogeneity and immunosuppressive ECM remodeling. The multimodal effects of ZR30, including inhibition of NOTCH signaling and angiogenesis, may facilitate blood vessel normalization, enhance T cell infiltration, and reduce immunosuppressive cytokine production. This positions ECM-targeting agents like ZR30 as promising candidates for combination therapies designed to disrupt the GSC-protective niche and prevent immune evasion (213). These findings establish the ECM as an active enhancer of NOTCH-driven immunosuppression and therapy resistance, indicating the potential of targeting ECM–NOTCH–immune interactions to improve GBM treatment.

## Epigenetic crosstalk regulating NOTCH-mediated immune evasion in glioma

Noting the established role of miRNAs in post-transcriptional regulation of NOTCH signaling, recent investigations have indicated the importance of epigenetic modifiers, including chromatin remodelers, histone-modifying enzymes, and methylation-dependent regulators, in forming NOTCH-mediated immune evasion in GBM (214). These epigenetic mechanisms influence NOTCH signaling either directly or through modulation of immune-related gene expression, and contribute to glioma stemness, immunosuppression, and therapy resistance (215). A key player in this process is the Polycomb group protein PCGF1, a component of the PRC1 complex, which substantially contributes to epigenetically enforcing NOTCH-dependent immune suppression. PCGF1 is upregulated in IDH-wildtype GBM and correlates with activation of NOTCH, PI3K-Akt, and JAK-STAT pathways. Tumors with high PCGF1 expression exhibit Th2-biased immunity and T cell depletion, a phenotype consistent with NOTCH-induced immunosuppression (216). PCGF1 is implicated in regulating several oncogenic signaling pathways, including NOTCH signaling, which governs cancer-related processes, tumor immunity, and glioma development. Through Polycomb-mediated silencing, PCGF1 represses antigen presentation and inflammatory cytokine genes, contributing to a hostile immune TME (216). Furthermore, WD40 repeat (WDR) domain-containing proteins are among the largest families in the human proteome that act as versatile scaffolds for protein–protein interactions (217) and maintain oncogenesis in various human cancers (218). WDR76, a WD40 domain chromatin-binding protein, is highly expressed in LGG. Elevated WDR76 expression is linked to more aggressive disease features and independently predicts poorer survival outcomes in LGG patients. This high expression, regulated by DNA methylation, leads to the upregulation of oncogenes such as PRC1 and NUSAP1, as well as activation of the NOTCH signaling pathway. Furthermore, WDR76 is implicated in LGG tumor immunity by facilitating the infiltration of immune cells, including M2 macrophages, and the expression of immune checkpoints, such as PDCD1 (which encodes PD-1) (219).

Beyond direct chromatin regulators, several oncogenic drivers have been identified as epigenetically regulated genes that affect NOTCH signaling and immune dynamics. CCDC34 is a coiled-coil domain-containing protein involved in tumor growth, apoptosis, and invasion, and has been implicated in the development and progression of several cancers (220). CCDC34 is overexpressed in LGGs and is governed by DNA methylation. It activates multiple oncogenic pathways, including NOTCH, PI3K-Akt, and HIF-1, contributing to glioma progression and potentially TAM-mediated immune evasion (221). Although the direct immunological role of CCDC34 is currently under investigation, its regulation and pathway enrichment analyses suggest that it may serve as a link between epigenetic regulation and immune responses. Similarly, the non-canonical NOTCH ligand DLL3, traditionally considered a cis-inhibitor of NOTCH receptors, is recognized for its context-dependent functions in glioma. DLL3 expression is epigenetically regulated, with its levels closely associated with promoter methylation and copy number variation (58, 59). Moreover, IDH mutations in gliomas are predictive of improved survival and are associated with epigenetic changes, including DNA hypermethylation, which alter gene expression and tumor behavior (222). In IDH1-mutant gliomas, cell surface NOTCH ligand DLL3 is upregulated and associated with improved prognosis and distinct immune infiltration patterns, suggesting a potentially immune-permissive function (86). Furthermore, DLL3 expression correlates with immune cell infiltration and negatively with primary cilia-related genes, indicating that DLL3 and the TME together influence LGG outcomes (59). These results demonstrate that DLL3 could serve as a subtype-specific biomarker in gliomas, with important implications for patient grouping (223). Moreover, Cao et al. demonstrated that the histone methyltransferase G9a (EHMT2) promotes NOTCH pathway activation by silencing FBXW7, a ubiquitin ligase that degrades NICD in GSCs. Through H3K9me2-dependent repression, G9a stabilizes NICD, driving sustained NOTCH activity, upregulating PD-L1, and downregulating MHC-I. These changes collectively suppress cytotoxic T cell infiltration and enhance MDSC and M2 macrophage expansion. Notably, inhibition of G9a reversed these effects and restored immune responsiveness, highlighting the therapeutic potential of targeting the G9a–FBXW7–NOTCH axis in combination with immune checkpoint inhibitors ^193^. In sum, Chromatin modifiers like G9a, PCGF1, and WDR76 promote NOTCH-driven immune evasion by suppressing antigen presentation and cytokines, while epigenetically regulated genes such as DLL3 and CCDC34 modulate NOTCH signaling and immune balance, highlighting the epigenetic–NOTCH–immune axis as a promising target to enhance antitumor immunity in glioma. **Table 4** shows a summary of the complex and interconnected epigenetic network that governs NOTCH signaling–immune crosstalk in gliomas.

**Table 4:** Epigenetic modulators and epigenetically regulated genes influencing NOTCH-driven immune evasion in glioma.

Gene/Protein	Epigenetic Role	Connection to NOTCH	Immune Impact	Therapeutic Relevance	Ref
G9a (EHMT2)	Histone methyltransferase (H3K9me2)	Silences FBXW7, stabilizes NICD	↑ PD-L1, ↓ MHC-I, ↑ MDSCs/M2	G9a inhibitors + ICIs	(224)
PCGF1	Polycomb group protein (PRC1 complex)	Reinforces NOTCH via gene silencing	↓ γδ T cells, Th2 skewing	Polycomb-targeted therapy	(216)
WDR76	Chromatin-associated modulator	Activates NOTCH–NUSAP1–PRC1 axis	↓ IL-15, ↑ PD-1, ↑ M2 TAMs	Potential immune-modulating target	(219)
CCDC34	Epigenetically regulated (DNA methylation)	Activates NOTCH, PI3K, HIF-1	Implicated in TAM polarization	Biomarker, NOTCH/HIF co-target	(221)
DLL3	Epigenetically regulated ligand (methylation, CNV)	Inhibits NOTCH (cis-inhibition)	Immune equilibrium regulator	Prognostic marker in IDH-mutant LGG	(58, 59)

FBXW7, F-box and WD repeat domain-containing 7; PD-L1, programmed death-ligand 1; MDSCs, myeloid-derived suppressor cells; TAMs, tumor-associated macrophages; NICD, NOTCH intracellular domain; PRC1, Polycomb repressive complex 1; CNV, copy number variation.

## Inflammation, coagulation, and NOTCH–immune crosstalk in gliomas

The TME is dominated by an excess of pro-angiogenic factors from cancerous, stromal, and immune cells, leading to abnormal blood vessel formation. These abnormal vessels create an inflammatory environment that promotes coagulation, thrombosis, and impaired blood flow, facilitating tumor growth and metastasis (225). Inflammation and coagulation are two distinct pathways that have evolved to function together, providing a strong defense against infection, damaged cells, and irritants, but when dysregulated or corrupted by tumors, like GBM, they can enhance tumor proliferation, invasion, dissemination, and survival (226, 227). Recent findings highlight that NOTCH signaling interacts with inflammation and coagulation pathways. In particular, the complement–coagulation cascade (CCC) has emerged as a novel partner of NOTCH signaling in promoting immune dysfunction and therapy resistance in glioma ^197^. Pro-inflammatory cytokines, such as TNFα, alter NOTCH signaling in vascular endothelial cells by decreasing NOTCH4 and increasing NOTCH2 expression, which leads to reduced NOTCH activity and promotes apoptosis. These changes, mediated through NF-κB and PI3-kinase pathways, suggest that inflammation-induced dysregulation of NOTCH contributes to endothelial and vascular dysfunction (55). There is evidence that specific targeting of these pathways may present significant potential for the future treatment of multiple tumor types, including GBM (228, 229).

Yang et al. developed a CCC-based risk signature including genes like F2R, SERPINA1, and C1Q, capable of grouping patients with LGG into high- and low-risk subtypes. The high-risk group exhibited co-activation of NOTCH, JAK-STAT, and PI3K-Akt signaling pathways, promoting stemness, immune suppression, and resistance to immunotherapy. Notably, C3AR1, a CCC effector, was implicated in MDSC recruitment and M2 macrophage polarization, phenocopying NOTCH-driven immunosuppression observed in GBM. Furthermore, the proteasome inhibitor MG-132, which is selectively effective in CCC-high LGGs, has been reported to inhibit both NF-κB and NOTCH signaling, thereby reversing thrombo-inflammatory responses. These data indicate the therapeutic potential of targeting the CCC–NOTCH axis in immune-refractory glioma subtypes (229).

## NOTCH mutations as predictive biomarkers for immunotherapy in GBM

Mutations affecting various components of the NOTCH signaling may play a role in the development of various malignancies, including GBM (230). An adeno-associated virus-mediated, autochthonous CRISPR genetic screen in GBM revealed frequent co-mutations of the NOTCH1 receptor and B2m, a main component of the MHC-I antigen presentation complex, suggesting a potential link between Notch signaling disruption and impaired immune recognition in glioma (132). NOTCH1 mutations are found in oligodendrogliomas, low-grade gliomas, and anaplastic astrocytoma that have an IDH mutation and 1p/19q codeletion. However, these mutations are infrequently seen in gliomas with wild-type IDH or in low-grade gliomas with an IDH mutation without 1p/19q codeletion (88).

Mutations in NOTCH receptor genes impact various regions of the protein with distinct functional consequences. Activating mutations are commonly found in the negative regulatory region, which regulates receptor cleavage, or in the C-terminal PEST domain, which controls NICD degradation. Mutations in the PEST domain often lead to prolonged NICD stability, resulting in extended or uncontrolled NOTCH signaling. These alterations lead to ligand-independent or sustained receptor activation (231). Conversely, inactivating NOTCH mutations primarily affect the extracellular epidermal growth factor-like repeats, disrupting ligand binding or causing truncated proteins. Such mutations often result in reduced expression of NOTCH target genes in glioma (11, 232). NOTCH2 protein expression is significantly higher in GBM compared to oligodendroglioma, where NOTCH2 loss-of-function mutations or deletions contribute to tumor development, suggesting that oligodendroglioma progression is linked to reduced or lost NOTCH2 activity (233).

Conversely, mutations in other NOTCH family members may exert antitumor effects through distinct mechanisms. NOTCH4 mutations are associated with significantly better outcomes, including higher objective response rates, durable clinical efficacy, and longer progression-free and overall survival outcomes, in individuals suffering from various cancers, including glioma. Multiomics analysis revealed that NOTCH4 mutations correlate with increased tumor immunogenicity and enhanced antitumor immune activity. These findings point to NOTCH4 mutation as a potential marker for predicting improved response to treatment with ICI (234). Tumors with NOTCH4 mutations were characterized by an intense immune micromilieu, including enhanced CD8^+^ T cell infiltration, high tumor mutational burden, and upregulated immune checkpoints, such as PD-1, PD-L1, and CTLA-4. This immunologically active profile is associated with improved survival following ICI therapy, independent of PD-L1 status or tumor mutational burden thresholds. While the link between NOTCH4 dysfunction and immune activation needs to be elucidated, it is hypothesized that loss-of-function mutations in NOTCH4 disrupt immunosuppressive NOTCH signaling circuits and enhance T cell recruitment and activation. These findings align with prior studies that indicate that NOTCH1 activity can suppress Th1-type inflammation (235), and with the growing use of synthetic NOTCH receptors in engineered immune cells to increase antigen-specific T cell responses (236, 237). In sum, NOTCH signaling mutations play a complex, context-dependent role in glioma. Certain mutations can enhance immune activation, making NOTCH a potential therapeutic target and a biomarker for guiding immunotherapy, while others promote immune evasion and tumor progression.

## Future perspectives

NOTCH signaling plays an important role in glioma tumorigenesis. Dysregulation of this pathway promotes tumor growth, stemness, and survival, while fostering an immunosuppressive microenvironment through interactions with immune cells. By modulating immune evasion and supporting tumor cell survival, NOTCH-immune crosstalk contributes to the aggressiveness and therapeutic resistance of gliomas. This review describes how aberrant activation of NOTCH receptors and ligands in tumor and stromal cells facilitates immune evasion via mechanisms like M2 macrophage polarization, Treg expansion, impaired antigen presentation, and reduced cytotoxic T cell infiltration. Furthermore, NOTCH signaling intersects with oncogenic pathways such as STAT3, HIF-1α, PI3K/AKT, and epigenetic regulators, collectively sustaining a therapy-resistant and immune-exclusive tumor phenotype. By maintaining stemness and promoting immune escape, NOTCH represents a promising therapeutic target in GBM. However, several key challenges remain unresolved. The context-dependent nature of NOTCH signaling, where both activation and suppression can produce immunosuppressive outcomes, complicates therapeutic targeting. In addition, the field lacks a clear mechanistic understanding of how tumor-intrinsic NOTCH activity integrates with systemic immune alterations (e.g., thymic atrophy and Treg bias), and why promising preclinical strategies have largely failed in clinical settings. Addressing these gaps will be essential for translating NOTCH biology into effective therapies. Despite advances in understanding NOTCH signaling and its mechanism of action, clinical translation of NOTCH-targeted therapies has been challenging. For instance, clinical trials evaluating first-generation pan-NOTCH inhibitors, such as RO4929097 (a GSI), have yielded minimal therapeutic benefit. In a Phase II study of 47 individuals with recurrent GBM, RO4929097 achieved a 6-month progression-free survival rate of just 4% and failed to inhibit neurosphere formation in 6 out of 7 resected patient tumor specimens (114). Moreover, the combination of RO4929097 and bevacizumab provided only limited long-term clinical activity (114). Earlier Phase 0/I clinical investigation showed pharmacodynamic evidence of NOTCH pathway inhibition, including reductions in NICD and CD133^+^ GSCs, but this molecular activity was not accompanied by substantial tumor control (111). The physical and metabolic microenvironment of the brain presents a significant challenge to effective NOTCH inhibition. First, the blood-brain barrier severely restricts the delivery of GSIs and other small molecules, leading to marked pharmacokinetic variability and heterogeneous drug penetration within different areas of the tumor *in vivo*. Consequently, therapeutic substances fail to achieve sustained and effective concentrations at the highly infiltrative tumor region. Second, first-generation inhibitors suffer from metabolic instability and auto-clearance (114). Specifically, RO4929097 has been shown to auto-induce its own metabolism via the cytochrome P450 CYP3A4 enzyme, rapidly reducing its systemic bioavailability and therapeutic window during continuous dosing regimens (114). Beyond pharmacology, the highly context-dependent, dual role of the NOTCH pathway within the TME poses a major biological challenge (165). While sustained NOTCH activity promotes an immunosuppressive niche, complete pathway suppression can paradoxically weaken anti-tumor immunity. Preclinical models demonstrated that the complete loss of NOTCH signaling impairs antigen presentation by downregulating MHC-I expression. Furthermore, complete NOTCH deficiency induces resistance to IFN-γ and triggers compensatory, immunosuppressive myeloid cell activation (165). This shows that effective clinical intervention should favor context-dependent and precisely regulated changes in signaling activity over complete or indiscriminate pathway inhibition. Together, these data point to a substantial preclinical–clinical disconnect and indicate that biological evidence supporting NOTCH as a therapeutic target should not be equated with clinically validated efficacy. Future investigations should focus on translating preclinical insights into clinically effective NOTCH–immune therapies while addressing treatment resistance, as outlined in the following suggestions:

1. Precision targeting strategies: Given the differential expression of NOTCH isoforms across distinct glioma subtypes, therapeutic interventions should carefully account for receptor specificity to enhance treatment efficacy and minimize adverse effects. NOTCH1 is significantly upregulated in the Classical and Proneural subtypes compared to the Mesenchymal subtype. Conversely, NOTCH2 and NOTCH3 exhibit higher expression in both the Classical and Mesenchymal subtypes but remain low in the Proneural subtype. NOTCH4 expression varies markedly between the Classical and Proneural subtypes. This indicates the complex and subtype-specific roles of NOTCH signaling in GBM biology (17). Furthermore, specific interactions between Notch and other pathways, such as STAT3 signaling in the mesenchymal GBM subtype, have been identified (85). Adapted approaches improve treatment effectiveness by specifically targeting GBM cells using modulators of distinct NOTCH subtypes. Future studies should define when NOTCH acts as a driver versus a regulator of immune balance, enabling more precise therapeutic modulation.
2. Predictive biomarkers for treatment efficacy: The identification and validation of robust predictive biomarkers are required for refining prognostic assessments and optimizing therapeutic strategies. For example, the NOTCH Score a quantitative metric derived from gene expression data measures Notch pathway activity in tumor tissues. Elevated NOTCH Scores are associated with poorer prognosis, potentially due to enhanced tumor immune evasion via upregulation of immune checkpoint molecules (62). Similarly, NOTCH4 mutations correlate with a less differentiated tumor phenotype and decreased overall survival in glioma patients, underscoring their potential as prognostic biomarkers for patient grouping and personalized treatment planning (238). Additionally, DLL methylation status functions as a key epigenetic biomarker in gliomas; aberrant methylation of DLL family members modulates gene expression, influencing tumor differentiation and Notch signaling dynamics. Integrating DLL3 methylation profiling with comprehensive NOTCH mutation analyses may offer a more nuanced molecular characterization of gliomas, thereby improving prognostic precision and facilitating targeted therapeutic interventions (136, 230). Collectively, the identification and validation of predictive biomarkers are essential for transitioning from broad NOTCH inhibition to a personalized, biomarker-guided treatment framework. Considering the molecular and cellular diversity of gliomas, a clinically meaningful patient selection strategy must integrate several biological layers, including: *(i)* stratification should incorporate NOTCH receptor/ligand expression profiles, distinguishing subtype-specific targets, such as NOTCH3 in classical/mesenchymal tumors or epigenetically regulated DLL3 in IDH-mutant gliomas; *(ii)* to capture dynamic pathway signaling, quantifying functional NOTCH activation via nuclear NICD localization is pivotal. This profile must be integrated with GSC-associated signatures (such as the transcriptomic NOTCH Score, the TME immune composition (including CD8^+^ T-cell infiltration, myeloid checkpoint landscapes, or loss-of-function NOTCH4 mutations), and hypoxia-related signatures that drive aggressive HIF-1α/NOTCH crosstalk; and *(iii)* aligning these features with relevant molecular subtypes and IDH status, particularly by combining DLL3 promoter methylation profiling with mutation typing, will establish a multi-dimensional stratification procedure. This framework will enable clinicians to design context-aware combination therapies designed to the unique microenvironmental and genetic landscape of each tumor.
3. Rational combination treatments: Future therapeutic success will likely depend on the development of rational combination regimens. Synergistic approaches that integrate NOTCH pathway inhibitors with immunotherapeutic modalities, such as PD-1/PD-L1 checkpoint blockade, CSF-1R antagonists, or engineered synNOTCH chimeric antigen receptor-T cells, have significant potential to overcome tumor immune evasion mechanisms and induce persistent anti-tumor responses (21, 239–241). Furthermore, concomitant targeting of key co-regulators of the NOTCH signaling pathway in glioma, including WNT5A, IRF7, and ZNF480, may concurrently disrupt cancer stemness properties and immunosuppressive signaling pathways and improve overall treatment outcomes (151, 242, 243). A key unresolved challenge is identifying which pathway combinations are most effective in specific tumor and immune contexts. Future therapeutic success will likely depend on the development of rational and context-dependent combination regimens. Synergistic approaches that integrate NOTCH pathway inhibitors with immunotherapeutic modalities, such as PD-1/PD-L1 checkpoint blockade, CSF-1R antagonists, or engineered synNOTCH chimeric antigen receptor-T cells, may have the potential to counter tumor immune evasion mechanisms and promote more durable anti-tumor responses (21, 228–230). Given the molecular and immunological heterogeneity of these tumors, the optimal combination is unlikely to be uniform across various forms of gliomas. Thus, biomarker-guided selection of combination partners and treatment strategies will be essential. Furthermore, concomitant targeting of key co-regulators of the NOTCH signaling pathway in glioma, including WNT5A, IRF7, and ZNF480, may concurrently disrupt cancer stemness properties and immunosuppressive signaling pathways and improve overall treatment outcomes (88, 146, 231). A key unresolved challenge is identifying which pathway combinations are most effective in specific tumor and immune contexts.
4. ECM remodeling strategies to enhance NOTCH-targeted therapies: As highlighted in the ECM section, the tumor matrix actively reinforces NOTCH-driven immune suppression and therapy resistance. However, the extent to which ECM remodeling can enhance immunotherapy responses remains unclear. Agents such as ZR30 demonstrate the potential to disrupt ECM–NOTCH interactions, but further work is needed to determine how these strategies influence immune cell infiltration and function *in vivo* (212, 213) (244). Understanding this interface represents an underexplored but promising therapeutic avenue.
5. Addressing mechanisms of resistance to NOTCH-targeted therapies: Investigate the molecular mechanisms underlying intrinsic and acquired resistance to NOTCH inhibitors in GBM. Understanding how tumor cells bypass NOTCH pathway inhibition, whether through compensatory signaling pathways, epigenetic alterations, or MTE interactions, will guide the development of next-generation inhibitors or combination strategies to overcome resistance. Several approaches targeting Notch-1 are under preclinical and clinical investigation as potential strategies to overcome GBM resistance. Investigations on these pathways may expand therapeutic options to combat this resistance (24, 245, 246).
6. Using computational models to improve Notch pathway targeting: Integrating approaches like artificial intelligence (AI) and machine learning into the study of Notch signaling enables the development of personalized, precise, and effective therapies. AI facilitates the elucidation of complex molecular mechanisms within the Notch pathway in specific tumor contexts, supporting more accurate therapeutic targeting (37). Moreover, AI-driven computational design tools can engineer synthetic molecules with the exact geometry and multivalency needed to activate Notch signaling. These advances can be broadly applied to optimize *in vitro* cell differentiation and accelerate the development of novel immunotherapies (247). The synergy between computational and biological sciences is transforming current approaches to GBM treatment and is instrumental in fully realizing the therapeutic potential of NOTCH pathway targeting (248).

## Conclusion

NOTCH signaling remains a highly compelling biological axis in glioma pathogenesis and plays integral roles in tumor stemness, immune evasion, and therapeutic resistance. However, a critical distinction must be made between this strong preclinical rationale and its clinically validated therapeutic efficacy, which remains limited to date. Rather than presenting broad, non-selective NOTCH inhibition as an immediately actionable clinical strategy, future translational efforts must shift toward context-aware, systems-level modulation. The field should prioritize isoform-, cell type-, and context-specific modulation of NOTCH signaling to reduce off-target toxicity and compensatory pathway activation. Integrating NOTCH targeting with biomarker-guided combination therapies, including co-targeting immune checkpoints, tumor-associated macrophages, or interacting developmental pathways, may provide a feasible strategy for the treatment of various forms of gliomas.

## Glossary

ADAM10/17: α-secretases
Ab: Antibody
Akt: Protein kinase B
B7-H4: B7 Homolog 4
CB-103: NOTCH transcriptional activation inhibitor
CCDC34: Coiled-coil domain-containing protein 34
CCL2: Chemokine (C-C motif) ligand 2
CHI3L1: GBM-associated glycoprotein
CNV: Copy number variation
CREB: cAMP response element-binding protein
CSF-1: Colony-stimulating factor 1
CSF-1R: Colony-stimulating factor 1 receptor
CXCL1: C-X-C motif chemokine ligand 1
DAPT: γ-secretase inhibitor drug
DLL1: Delta-like ligand 1
DLL3: Delta-like ligand 3
DLL4: Delta-like ligand 4
DP thymocytes: Double-positive thymocytes
ECM: Extracellular matrix
EFEMP1: Epidermal growth factor-containing fibulin-like extracellular matrix protein 1
EZH2: Enhancer of zeste homolog 2
FBXW7: F-box and WD repeat domain-containing 7
FOXM1: Forkhead box protein M1
Forskolin: Adenylate cyclase activator
GBM: Glioblastoma
GI: Gastrointestinal
GSC: Glioma stem cell
GSI: γ-secretase inhibitor
HES1: Hairy and enhancer of split 1
HES5: Hairy and enhancer of split 5
HES6: Hairy and enhancer of split 6
HEY1: Hes-related family BHLH transcription factor with YRPW motif 1
HEY2: Hes-related family BHLH transcription factor with YRPW motif 2
HIF-1α: Hypoxia-inducible factor 1-alpha
HO-1: Heme oxygenase 1
ICI: Immune checkpoint inhibitor
IDH: Isocitrate dehydrogenase
IDH1: Isocitrate dehydrogenase 1
IKKα/IKKβ: Inhibitor of nuclear factor kappa-B kinase subunit alpha/beta
IFN-γ: interferon-γ
IL-10: Interleukin-10
IL-12: Interleukin-12
IL-23: Interleukin-23
INCB3619: Dual ADAM10/17 inhibitor
IRF7: Interferon regulatory factor 7
JAG1: Jagged1
JAG2: Jagged2
JAK: Janus kinase
JAK/STAT: Janus kinase/signal transducer and activator of transcription
LIF: Leukemia inhibitory factor
MAML: Mastermind-like transcriptional coactivator
MDSCs: Myeloid-derived suppressor cells
MHC-I: Major histocompatibility complex class I
MMP2: Matrix metalloproteinase-2
MMPs: Matrix metalloproteinases
MRK003: γ-secretase inhibitor drug
NF-κB: Nuclear factor kappa-light-chain-enhancer of activated B cells
NICD: NOTCH intracellular domain
NUP205: Nucleoporin 205
NUSAP1: Nucleolar and spindle-associated protein 1
PD-L1: Programmed death-ligand 1
PI3K: Phosphoinositide 3-kinase
PKA: Protein kinase A
PRC1: Polycomb repressive complex 1
RBP-J: Recombination signal binding protein for immunoglobulin kappa J region
REST: RE1-silencing transcription factor
RO4929097: γ-secretase inhibitor drug
Ror1: Receptor tyrosine kinase-like orphan receptor 1
SEMA3B: Semaphorin 3B
STAT3: Signal transducer and activator of transcription 3
SUZ12: Suppressor of zeste 12
TAMs: Tumor-associated macrophages
TGF-β: Transforming growth factor-beta
TME: Tumor microenvironment
TMZ: Temozolomide
TNF-α: Tumor necrosis factor-alpha
TP1: Tumor phenotype subtype 1
Tregs: Regulatory T cells
VDR: Vitamin D receptor
VEGF: Vascular endothelial growth factor
WDR76: WD repeat domain 76
WNT5A: Wnt family member 5A
YAP1: Yes-associated protein 1
YKL-40: GBM-associated glycoprotein
ZNF480: Zinc finger protein 480
ZR30: Engineered fibulin-3 derivative
mTOR: Mammalian target of rapamycin
oHSV: Oncolytic herpes simplex virus.

## References

[1] Ohgaki H Kleihues P . Epidemiology and etiology of gliomas. Acta Neuropathol. (2005) 109:93–108. doi: 10.1007/s00401-005-0991-y 15685439

[2] Wen PY Kesari S . Malignant gliomas in adults. N Engl J Med. (2008) 359:492–507. doi: 10.1056/nejmra0708126 18669428

[3] Louis DN Perry A Wesseling P Brat DJ Cree IA Figarella-Branger D . The 2021 WHO classification of tumors of the central nervous system: a summary. Neuro Oncol. (2021) 23:1231–51. doi: 10.1093/neuonc/noab106 34185076 PMC8328013

[4] Delgado-Lopez PD Corrales-Garcia EM . Survival in glioblastoma: a review on the impact of treatment modalities. Clin Transl Oncol. (2016) 18:1062–71. doi: 10.1007/s12094-016-1497-x 26960561

[5] Nayak L Reardon DA . High-grade gliomas. CONTINUUM: Lifelong Learn Neurol. (2017) 23:1548–63. doi: 10.1212/con.0000000000000554 29200110

[6] Bikfalvi A da Costa CA Avril T Barnier JV Bauchet L Brisson L . Challenges in glioblastoma research: focus on the tumor microenvironment: (Trends in Cancer, 9:1 p:9-27, 2023). Trends Cancer. (2023) 9:692. doi: 10.1016/j.trecan.2023.02.006 36400694

[7] Charles NA Holland EC Gilbertson R Glass R Kettenmann H . The brain tumor microenvironment. Glia. (2011) 59:1169–80. doi: 10.1002/glia.21264 21446047

[8] Fehervari Z . Glioma immune evasion. Nat Immunol. (2017) 18:487. doi: 10.1038/ni.3736 28418388

[9] Gieryng A Pszczolkowska D Walentynowicz KA Rajan WD Kaminska B . Immune microenvironment of gliomas. Lab Invest. (2017) 97:498–518. doi: 10.1038/labinvest.2017.19 28287634

[10] Aster JC Pear WS Blacklow SC . The varied roles of Notch in cancer. Annu Rev Pathol. (2017) 12:245–75. doi: 10.1146/annurev-pathol-052016-100127 27959635 PMC5933931

[11] Parmigiani E Taylor V Giachino C . Oncogenic and tumor-suppressive functions of NOTCH signaling in glioma. Cells. (2020) 9:2304. doi: 10.3390/cells9102304 33076453 PMC7602630

[12] Fan X Khaki L Zhu TS Soules ME Talsma CE Gul N . NOTCH pathway blockade depletes CD133-positive glioblastoma cells and inhibits growth of tumor neurospheres and xenografts. Stem Cells. (2010) 28:5–16. doi: 10.1002/stem.254 PMC2878196 19904829

[13] Cenciarelli C Marei HE Zonfrillo M Casalbore P Felsani A Giannetti S . The interference of Notch1 target Hes1 affects cell growth, differentiation and invasiveness of glioblastoma stem cells through modulation of multiple oncogenic targets. Oncotarget. (2017) 8:17873–86. doi: 10.18632/oncotarget.15013 28157712 PMC5392293

[14] Han X Qin H Lu Y Chen H Yuan Z Zhang Y . Post-translational modifications: The potential ways for killing cancer stem cells. Heliyon. (2024) 10:e34015. doi: 10.1016/j.heliyon.2024.e34015 39092260 PMC11292267

[15] Stockhausen M-T Kristoffersen K Poulsen HS . The functional role of Notch signaling in human gliomas. Neuro-oncology. (2010) 12:199–211. doi: 10.1093/neuonc/nop022 PMC2940575 20150387

[16] Kipper FC Kieran MW Thomas A Panigrahy D . Notch signaling in malignant gliomas: Supporting tumor growth and the vascular environment. Cancer Metastasis Rev. (2022) 41:737–47. doi: 10.1007/s10555-022-10041-7 35624227

[17] Zheng ZQ Zhang GG Yuan GQ Hao JH Nie QQ Zheng MC . Development and validation of an immune infiltration/tumor proliferation-related Notch3 nomogram for predicting survival in patients with primary glioblastoma. Front Genet. (2023) 14:1148126. doi: 10.3389/fgene.2023.1148126 37284062 PMC10240236

[18] Meurette O . Shaping of the tumor microenvironment by Notch signaling. Adv Exp Med Biol. (2020) 1223:1–16. doi: 10.1007/978-3-030-35582-1_1 32030682

[19] Hossain F Majumder S Ucar DA Rodriguez PC Golde TE Minter LM . Notch signaling in myeloid cells as a regulator of tumor immune responses. Front Immunol. (2018) 9:1288. doi: 10.3389/fimmu.2018.01288 29915603 PMC5994797

[20] Janghorban M Xin L Rosen JM Zhang XH-F . Notch signaling as a regulator of the tumor immune response: to target or not to target? Front Immunol. (2018) 9:1649. doi: 10.3389/fimmu.2018.01649 30061899 PMC6055003

[21] Li X Yan X Wang Y Kaur B Han H Yu J . The Notch signaling pathway: a potential target for cancer immunotherapy. J Hematol Oncol. (2023) 16:45. doi: 10.1186/s13045-023-01439-z 37131214 PMC10155406

[22] Zhu TS Costello MA Talsma CE Flack CG Crowley JG Hamm LL . Endothelial cells create a stem cell niche in glioblastoma by providing NOTCH ligands that nurture self-renewal of cancer stem-like cells. Cancer Res. (2011) 71:6061–72. doi: 10.1158/0008-5472.can-10-4269 21788346 PMC3355476

[23] Qiu XX Chen L Wang CH Lin ZX Chen BJ You N . The vascular Notch ligands Delta-Like Ligand 4 (DLL4) and Jagged1 (JAG1) have opposing correlations with microvascularization but a uniform prognostic effect in primary glioblastoma: a preliminary study. World Neurosurg. (2016) 88:447–58. doi: 10.1016/j.wneu.2015.10.058 26546995

[24] Gersey Z Osiason AD Bloom L Shah S Thompson JW Bregy A . Therapeutic targeting of the Notch pathway in glioblastoma multiforme. World Neurosurg. (2019) 131:252–63:e252. doi: 10.1016/j.wneu.2019.07.180 31376551

[25] Portela M Venkataramani V Fahey-Lozano N Seco E Losada-Perez M Winkler F . Glioblastoma cells vampirize WNT from neurons and trigger a JNK/MMP signaling loop that enhances glioblastoma progression and neurodegeneration. PloS Biol. (2019) 17:e3000545. doi: 10.1371/journal.pbio.3000545 31846454 PMC6917273

[26] Wang S Gu S Chen J Yuan Z Liang P Cui H . Mechanism of Notch signaling pathway in malignant progression of glioblastoma and targeted therapy. Biomolecules. (2024) 14:480. doi: 10.3390/biom14040480 38672496 PMC11048644

[27] Zhang H Hang W Jing Z Liu B Wang X Li Y . The role of Notch signaling pathway in cancer: mechanistic insights, therapeutic potential, and clinical progress. Front Immunol. (2025) 16:1567524. doi: 10.3389/fimmu.2025.1567524 40630953 PMC12235416

[28] Andersson ER Sandberg R Lendahl U . Notch signaling: simplicity in design, versatility in function. Development. (2011) 138:3593–612. doi: 10.1242/dev.063610 21828089

[29] Guruharsha KG Kankel MW Artavanis-Tsakonas S . The Notch signalling system: recent insights into the complexity of a conserved pathway. Nat Rev Genet. (2012) 13:654–66. doi: 10.1038/nrg3272 22868267 PMC4369923

[30] Greenwald I Kovall R . Notch signaling: genetics and structure. WormBook. (2013), 1–28. doi: 10.1895/wormbook.1.10.2 23355521 PMC5402211

[31] Zhou B Lin W Long Y Yang Y Zhang H Wu K . Notch signaling pathway: architecture, disease, and therapeutics. Signal Transduct Target Ther. (2022) 7:95. doi: 10.1038/s41392-022-00934-y 35332121 PMC8948217

[32] Bazzoni R Bentivegna A . Role of Notch signaling pathway in glioblastoma pathogenesis. Cancers (Basel). (2019) 11:292. doi: 10.3390/cancers11030292 30832246 PMC6468848

[33] Nueda ML Baladron V . Mammalian NOTCH receptor activation and signaling protocols. Methods Mol Biol. (2022) 2472:67–82. doi: 10.1007/978-1-0716-2201-8_7 35674893

[34] Faraji N Ebadpour N Abavisani M Gorji A . Unlocking hope: Therapeutic advances and approaches in modulating the Wnt pathway for neurodegenerative diseases. Mol Neurobiol. (2025) 62:3630–52. doi: 10.1007/s12035-024-04462-4 PMC11790780 39313658

[35] Sanders PG Munoz-Descalzo S Balayo T Wirtz-Peitz F Hayward P Arias AM . Ligand-independent traffic of Notch buffers activated Armadillo in Drosophila. PloS Biol. (2009) 7:e1000169. doi: 10.1371/journal.pbio.1000169 19668359 PMC2716527

[36] Sanalkumar R Dhanesh SB James J . Non-canonical activation of Notch signaling/target genes in vertebrates. Cell Mol Life Sci. (2010) 67:2957–68. doi: 10.1007/s00018-010-0391-x 20458516 PMC11115867

[37] Shi Q Xue C Zeng Y Yuan X Chu Q Jiang S . Notch signaling pathway in cancer: from mechanistic insights to targeted therapies. Signal Transduct Target Ther. (2024) 9:128. doi: 10.1038/s41392-024-01828-x 38797752 PMC11128457

[38] Kwon C Cheng P King IN Andersen P Shenje L Nigam V . Notch post-translationally regulates beta-catenin protein in stem and progenitor cells. Nat Cell Biol. (2011) 13:1244–51. doi: 10.1038/ncb2313 21841793 PMC3187850

[39] Bhat V Sun YJ Weger S Raouf A . Notch-induced expression of FZD7 requires noncanonical NOTCH3 signaling in human breast epithelial cells. Stem Cells Dev. (2016) 25:522–39. doi: 10.1089/scd.2015.0315 26847503

[40] Jin S Mutvei AP Chivukula IV Andersson ER Ramskold D Sandberg R . Non-canonical Notch signaling activates IL-6/JAK/STAT signaling in breast tumor cells and is controlled by p53 and IKKalpha/IKKbeta. Oncogene. (2013) 32:4892–902. doi: 10.1038/onc.2012.517 PMC3795477 23178494

[41] Calzavara E Chiaramonte R Cesana D Basile A Sherbet GV Comi P . Reciprocal regulation of Notch and PI3K/Akt signalling in T-ALL cells *in vitro*. J Cell Biochem. (2008) 103:1405–12. doi: 10.1002/jcb.21527 17849443

[42] Perumalsamy LR Nagala M Banerjee P Sarin A . A hierarchical cascade activated by non-canonical Notch signaling and the mTOR-Rictor complex regulates neglect-induced death in mammalian cells. Cell Death Differ. (2009) 16:879–89. doi: 10.1038/cdd.2009.20 19265851

[43] Lee KS Wu Z Song Y Mitra SS Feroze AH Cheshier SH . Roles of PINK1, mTORC2, and mitochondria in preserving brain tumor-forming stem cells in a noncanonical Notch signaling pathway. Genes Dev. (2013) 27:2642–7. doi: 10.1101/gad.225169.113 24352421 PMC3877754

[44] Xing Z Sun L Guo W . Elevated expression of Notch-1 and EGFR induced apoptosis in glioblastoma multiforme patients. Clin Neurol Neurosurg. (2015) 131:54–8. doi: 10.1016/j.clineuro.2015.01.018 25704190

[45] Han N Hu G Shi L Long G Yang L Xi Q . Notch1 ablation radiosensitizes glioblastoma cells. Oncotarget. (2017) 8:88059. doi: 10.18632/oncotarget.21409 PMC5675693 29152141

[46] Man J Yu X Huang H Zhou W Xiang C Huang H . Hypoxic induction of vasorin regulates Notch1 turnover to maintain glioma stem-like cells. Cell Stem Cell. (2018) 22:104–18:e106. doi: 10.1016/j.stem.2017.10.005 29198941 PMC5756127

[47] Dell'Albani P Rodolico M Pellitteri R Tricarichi E Torrisi SA D'Antoni S . Differential patterns of NOTCH1–4 receptor expression are markers of glioma cell differentiation. Neuro-oncology. (2014) 16:204–16. doi: 10.1093/neuonc/not168 PMC3895382 24305720

[48] Alqudah MA Agarwal S Al-Keilani MS Sibenaller ZA Ryken TC Assem M . NOTCH3 is a prognostic factor that promotes glioma cell proliferation, migration and invasion via activation of CCND1 and EGFR. PloS One. (2013) 8:e77299. doi: 10.1371/journal.pone.0077299 24143218 PMC3797092

[49] Alqudah MAY . The Oncogenic Role and the Prognostic Value of NOTCH3 Gene in Human Malignant Glioma. Iowa City, Iowa: The University of Iowa (2013). doi: 10.17077/etd.lbpsvake

[50] Tchorz J Tome M Cloëtta D Sivasankaran B Grzmil M Huber R . Constitutive Notch2 signaling in neural stem cells promotes tumorigenic features and astroglial lineage entry. Cell Death Dis. (2012) 3:e325-e325. doi: 10.1038/cddis.2012.65 22717580 PMC3388237

[51] Jubb AM Browning L Campo L Turley H Steers G Thurston G . Expression of vascular Notch ligands Delta‐like 4 and Jagged‐1 in glioblastoma. Histopathology. (2012) 60:740–7. doi: 10.1111/j.1365-2559.2011.04138.x 22296176

[52] Zhang J Chen Y Qiu X Tang W Zhang J Huang J . The vascular delta-like ligand-4 (DLL4)-Notch4 signaling correlates with angiogenesis in primary glioblastoma: an immunohistochemical study. Tumor Biol. (2016) 37:3797–805. doi: 10.1007/s13277-015-4202-8 26472724

[53] Oon CE Bridges E Sheldon H Sainson RC Jubb A Turley H . Role of Delta-like 4 in Jagged1-induced tumour angiogenesis and tumour growth. Oncotarget. (2017) 8:40115. doi: 10.18632/oncotarget.16969 28445154 PMC5522274

[54] Dell'albani P Rodolico M Pellitteri R Tricarichi E Torrisi SA D'Antoni S . Differential patterns of NOTCH1-4 receptor expression are markers of glioma cell differentiation. Neuro Oncol. (2014) 16:204–16. doi: 10.1093/neuonc/not168 PMC3895382 24305720

[55] Quillard T Devalliere J Coupel S Charreau B . Inflammation dysregulates Notch signaling in endothelial cells: implication of Notch2 and Notch4 to endothelial dysfunction. Biochem Pharmacol. (2010) 80:2032–41. doi: 10.1016/j.bcp.2010.07.010 20643108

[56] Jubb AM Browning L Campo L Turley H Steers G Thurston G . Expression of vascular Notch ligands Delta-like 4 and Jagged-1 in glioblastoma. Histopathology. (2012) 60:740–7. doi: 10.1111/j.1365-2559.2011.04138.x 22296176

[57] Spino M Kurz SC Chiriboga L Serrano J Zeck B Sen N . Cell surface notch ligand dll3 is a therapeutic target in isocitrate dehydrogenase–mutant glioma. Clin Cancer Res. (2019) 25:1261–71. doi: 10.1158/1078-0432.ccr-18-2312 30397180 PMC7365589

[58] Maimaiti A Wang X Hao Y Jiang L Shi X Pei Y . Integrated gene expression and methylation analyses identify DLL3 as a biomarker for prognosis of malignant glioma. J Mol Neurosci. (2021) 71:1622–35. doi: 10.1007/s12031-021-01817-7 33713320

[59] Noor H Whittaker S McDonald KL . DLL3 expression and methylation are associated with lower-grade glioma immune microenvironment and prognosis. Genomics. (2022) 114:110289. doi: 10.1016/j.ygeno.2022.110289 35124175

[60] Li JL Sainson RC Oon CE Turley H Leek R Sheldon H . DLL4-Notch signaling mediates tumor resistance to anti-VEGF therapy *in vivo*. Cancer Res. (2011) 71:6073–83. doi: 10.1158/0008-5472.can-11-1704 21803743

[61] Otani Y Yoo JY Lewis CT Chao S Swanner J Shimizu T . NOTCH-induced MDSC recruitment after oHSV virotherapy in CNS cancer models modulates antitumor immunotherapy. Clin Cancer Res. (2022) 28:1460–73. doi: 10.1158/1078-0432.ccr-21-2347 35022322 PMC8976724

[62] Shi B Ge F Cai L Yang Y Guo X Wu R . Significance of NotchScore and JAG1 in predicting prognosis and immune response of low-grade glioma. Front Immunol. (2023) 14:1247288. doi: 10.3389/fimmu.2023.1247288 38022677 PMC10679421

[63] Nakamura Y Sakakibara S Miyata T Ogawa M Shimazaki T Weiss S . The bHLH gene hes1 as a repressor of the neuronal commitment of CNS stem cells. J Neurosci. (2000) 20:283–93. doi: 10.1523/jneurosci.20-01-00283.2000 10627606 PMC6774123

[64] Radtke F MacDonald HR Tacchini-Cottier F . Regulation of innate and adaptive immunity by Notch. Nat Rev Immunol. (2013) 13:427–38. doi: 10.1038/nri3445 23665520

[65] Masek J Andersson ER . The developmental biology of genetic Notch disorders. Development. (2017) 144:1743–69. doi: 10.1242/dev.148007 28512196

[66] Paniri A Hosseini MM Amjadi-Moheb F Tabaripour R Soleimani E Langroudi MP . The epigenetics orchestra of Notch signaling: a symphony for cancer therapy. Epigenomics. (2023) 15:1337–58. doi: 10.2217/epi-2023-0270 38112013

[67] Kuintzle R Santat LA Elowitz MB . Diversity in Notch ligand-receptor signaling interactions. Elife. (2025) 12. doi: 10.7554/elife.91422.3 PMC11698495 39751380

[68] Yu J Canalis E . Notch and the regulation of osteoclast differentiation and function. Bone. (2020) 138:115474. doi: 10.1016/j.bone.2020.115474 32526405 PMC7423683

[69] Hu B Phan SH . Notch in fibrosis and as a target of anti-fibrotic therapy. Pharmacol Res. (2016) 108:57–64. doi: 10.1016/j.phrs.2016.04.010 27107790 PMC5074914

[70] Weng AP Ferrando AA Lee W Morris JP Silverman LB Sanchez-Irizarry C . Activating mutations of NOTCH1 in human T cell acute lymphoblastic leukemia. Science. (2004) 306:269–71. doi: 10.1126/science.1102160 15472075

[71] Dotto GP . Notch tumor suppressor function. Oncogene. (2008) 27:5115–23. doi: 10.1038/onc.2008.225 18758480 PMC2747622

[72] Purow BW Haque RM Noel MW Su Q Burdick MJ Lee J . Expression of Notch-1 and its ligands, Delta-like-1 and Jagged-1, is critical for glioma cell survival and proliferation. Cancer Res. (2005) 65:2353–63. doi: 10.1158/0008-5472.can-04-1890 15781650

[73] Zhang XP Zheng G Zou L Liu HL Hou LH Zhou P . Notch activation promotes cell proliferation and the formation of neural stem cell-like colonies in human glioma cells. Mol Cell Biochem. (2008) 307:101–8. doi: 10.1007/s11010-007-9589-0 17849174

[74] Hai L Zhang C Li T Zhou X Liu B Li S . Notch1 is a prognostic factor that is distinctly activated in the classical and proneural subtype of glioblastoma and that promotes glioma cell survival via the NF-κB (p65) pathway. Cell Death Dis. (2018) 9:158. doi: 10.1038/s41419-017-0119-z 29410396 PMC5833555

[75] Xu J Jin XL Shen H Chen XW Chen J Huang H . NOTCH3 as a prognostic biomarker and its correlation with immune infiltration in gastrointestinal cancers. Sci Rep. (2024) 14:14327. doi: 10.1038/s41598-024-65036-x 38906903 PMC11192884

[76] Sarkar S Mirzaei R Zemp FJ Wei W Senger DL Robbins SM . Activation of NOTCH signaling by Tenascin-C promotes growth of human brain tumor-initiating cells. Cancer Res. (2017) 77:3231–43. doi: 10.1158/0008-5472.can-16-2171 28416488

[77] Guichet P-O Guelfi S Teigell M Hoppe L Bakalara N Bauchet L . Notch1 stimulation induces a vascularization switch with pericyte-like cell differentiation of glioblastoma stem cells. Stem Cells. (2015) 33:21–34. doi: 10.1002/stem.1767 24898819

[78] Zheng H Ying H Yan H Kimmelman AC Hiller DJ Chen A-J . p53 and Pten control neural and glioma stem/progenitor cell renewal and differentiation. Nature. (2008) 455:1129–34. doi: 10.1038/nature07443 18948956 PMC4051433

[79] Zhang X Chen T Zhang J Mao Q Li S Xiong W . Notch1 promotes glioma cell migration and invasion by stimulating β‐catenin and NF‐κB signaling via AKT activation. Cancer Sci. (2012) 103:181–90. doi: 10.1111/j.1349-7006.2011.02154.x 22093097

[80] Qiang L Wu T Zhang H Lu N Hu R Wang Y . HIF-1α is critical for hypoxia-mediated maintenance of glioblastoma stem cells by activating Notch signaling pathway. Cell Death Differ. (2012) 19:284–94. doi: 10.1038/cdd.2011.95 PMC3263503 21818118

[81] Ruan J Lou S Dai Q Mao D Ji J Sun X . Tumor suppressor miR-181c attenuates proliferation, invasion, and self-renewal abilities in glioblastoma. Neuroreport. (2015) 26:66–73. doi: 10.1097/wnr.0000000000000302 25494473

[82] Tsung AJ Guda MR Asuthkar S Labak CM Purvis IJ Lu Y . Methylation regulates HEY1 expression in glioblastoma. Oncotarget. (2017) 8:44398–409. doi: 10.18632/oncotarget.17897 28574840 PMC5546488

[83] Di Bari M Bevilacqua V De Jaco A Laneve P Piovesana R Trobiani L . Mir-34a-5p mediates cross-talk between M2 muscarinic receptors and Notch-1/EGFR pathways in U87MG glioblastoma cells: implication in cell proliferation. Int J Mol Sci. (2018) 19. doi: 10.3390/ijms19061631 29857516 PMC6032387

[84] Huang YC Lin SJ Shih HY Chou CH Chu HH Chiu CC . Epigenetic regulation of NOTCH1 and NOTCH3 by KMT2A inhibits glioma proliferation. Oncotarget. (2017) 8:63110–20. doi: 10.18632/oncotarget.18668 28968975 PMC5609907

[85] Cheng W Zhang C Ren X Jiang Y Han S Liu Y . Bioinformatic analyses reveal a distinct Notch activation induced by STAT3 phosphorylation in the mesenchymal subtype of glioblastoma. J Neurosurg. (2017) 126:249–59. doi: 10.3171/2015.11.jns15432 26967788

[86] Spino M Kurz SC Chiriboga L Serrano J Zeck B Sen N . Cell surface Notch ligand DLL3 is a therapeutic target in isocitrate dehydrogenase-mutant glioma. Clin Cancer Res. (2019) 25:1261–71. doi: 10.1158/1078-0432.ccr-18-2312 30397180 PMC7365589

[87] Bettegowda C Agrawal N Jiao Y Sausen M Wood LD Hruban RH . Mutations in CIC and FUBP1 contribute to human oligodendroglioma. Science. (2011) 333:1453–5. doi: 10.1126/science.1210557 21817013 PMC3170506

[88] Brat D Verhaak R Aldape K Yung W Salama S Cooper L . Comprehensive, integrative genomic analysis of diffuse lower-grade gliomas. N Engl J Med. (2015) 2481:10. 10.1056/NEJMoa1402121 PMC4530011 26061751

[89] Suzuki H Aoki K Chiba K Sato Y Shiozawa Y Shiraishi Y . Mutational landscape and clonal architecture in grade II and III gliomas. Nat Genet. (2015) 47:458–68. doi: 10.1038/ng.3273 25848751

[90] Bai H Harmancı AS Erson-Omay EZ Li J Coşkun S Simon M . Integrated genomic characterization of IDH1-mutant glioma malignant progression. Nat Genet. (2016) 48:59–66. doi: 10.1038/ng.3457 PMC4829945 26618343

[91] Halani SH Yousefi S Velazquez Vega J Rossi MR Zhao Z Amrollahi F . Multi-faceted computational assessment of risk and progression in oligodendroglioma implicates NOTCH and PI3K pathways. NPJ Precis Oncol. (2018) 2:24. doi: 10.1038/s41698-018-0067-9 30417117 PMC6219505

[92] Giachino C Boulay J-L Ivanek R Alvarado A Tostado C Lugert S . A tumor suppressor function for notch signaling in forebrain tumor subtypes. Cancer Cell. (2015) 28:730–42. doi: 10.1016/j.ccell.2015.10.008 26669487

[93] Barone C Buccarelli M Alessandrini F Pagin M Rigoldi L Sambruni I . Sox2‐dependent maintenance of mouse oligodendroglioma involves the Sox2‐mediated downregulation of Cdkn2b, Ebf1, Zfp423, and Hey2. Glia. (2021) 69:579–93. doi: 10.1002/glia.23914 32975900

[94] Somasundaram K Reddy SP Vinnakota K Britto R Subbarayan M Nambiar S . Upregulation of ASCL1 and inhibition of Notch signaling pathway characterize progressive astrocytoma. Oncogene. (2005) 24:7073–83. doi: 10.1038/sj.onc.1208865 16103883

[95] Blomfield IM Rocamonde B Masdeu MM Mulugeta E Vaga S van den Berg DL . Id4 promotes the elimination of the pro-activation factor Ascl1 to maintain quiescence of adult hippocampal stem cells. Elife. (2019) 8:e48561. doi: 10.7554/elife.48561 31552825 PMC6805120

[96] Narayanan A Gagliardi F Gallotti AL Mazzoleni S Cominelli M Fagnocchi L . The proneural gene ASCL1 governs the transcriptional subgroup affiliation in glioblastoma stem cells by directly repressing the mesenchymal gene NDRG1. Cell Death Differ. (2019) 26:1813–31. doi: 10.1038/s41418-018-0248-7 PMC6748080 30538287

[97] D'Amico M De Amicis F . Aberrant Notch signaling in gliomas: a potential landscape of actionable converging targets for combination approach in therapies resistance. Cancer Drug Resist. (2022) 5:939–53. doi: 10.20517/cdr.2022.46 PMC9771760 36627893

[98] Akil A Gutierrez-Garcia AK Guenter R Rose JB Beck AW Chen H . Notch signaling in vascular endothelial cells, angiogenesis, and tumor progression: an update and prospective. Front Cell Dev Biol. (2021) 9:642352. doi: 10.3389/fcell.2021.642352 33681228 PMC7928398

[99] Harwood DSL Pedersen V Bager NS Schmidt AY Stannius TO Areskeviciute A . Glioblastoma cells increase expression of notch signaling and synaptic genes within infiltrated brain tissue. Nat Commun. (2024) 15:7857. doi: 10.1038/s41467-024-52167-y 39251578 PMC11385527

[100] Li D Masiero M Banham AH Harris AL . The notch ligand JAGGED1 as a target for anti-tumor therapy. Front Oncol. (2014) 4:254. doi: 10.3389/fonc.2014.00254 25309874 PMC4174884

[101] Brooks LJ Parrinello S . Vascular regulation of glioma stem-like cells: a balancing act. Curr Opin Neurobiol. (2017) 47:8–15. doi: 10.1016/j.conb.2017.06.008 28732340

[102] Lin H Liu C Hu A Zhang D Yang H Mao Y . Understanding the immunosuppressive microenvironment of glioma: mechanistic insights and clinical perspectives. J Hematol Oncol. (2024) 17:31. doi: 10.1186/s13045-024-01544-7 38720342 PMC11077829

[103] Tang J Karbhari N Campian JL . Therapeutic targets in glioblastoma: molecular pathways, emerging strategies, and future directions. Cells. (2025) 14. doi: 10.3390/cells14070494 40214448 PMC11988183

[104] Chen J Kesari S Rooney C Strack PR Chen J Shen H . Inhibition of notch signaling blocks growth of glioblastoma cell lines and tumor neurospheres. Genes Cancer. (2010) 1:822–35. doi: 10.1177/1947601910383564 21127729 PMC2994256

[105] Hovinga KE Shimizu F Wang R Panagiotakos G Van Der Heijden M Moayedpardazi H . Inhibition of notch signaling in glioblastoma targets cancer stem cells via an endothelial cell intermediate. Stem Cells. (2010) 28:1019–29. doi: 10.1002/stem.429 20506127 PMC5532884

[106] Kristoffersen K Villingshoj M Poulsen HS Stockhausen MT . Level of Notch activation determines the effect on growth and stem cell-like features in glioblastoma multiforme neurosphere cultures. Cancer Biol Ther. (2013) 14:625–37. doi: 10.4161/cbt.24595 23792644 PMC3742492

[107] Saito N Fu J Zheng S Yao J Wang S Liu DD . A high Notch pathway activation predicts response to gamma secretase inhibitors in proneural subtype of glioma tumor-initiating cells. Stem Cells. (2014) 32:301–12. doi: 10.1002/stem.1528 24038660 PMC3947402

[108] Tanaka S Nakada M Yamada D Nakano I Todo T Ino Y . Strong therapeutic potential of gamma-secretase inhibitor MRK003 for CD44-high and CD133-low glioblastoma initiating cells. J Neuro-Oncol. (2015) 121:239–50. doi: 10.1007/s11060-014-1630-z 25293440

[109] Yahyanejad S King H Iglesias VS Granton PV Barbeau LM van Hoof SJ . NOTCH blockade combined with radiation therapy and temozolomide prolongs survival of orthotopic glioblastoma. Oncotarget. (2016) 7:41251–64. doi: 10.18632/oncotarget.9275 27183910 PMC5173056

[110] Hiddingh L Tannous BA Teng J Tops B Jeuken J Hulleman E . EFEMP1 induces gamma-secretase/Notch-mediated temozolomide resistance in glioblastoma. Oncotarget. (2014) 5:363–74. doi: 10.18632/oncotarget.1620 PMC3964213 24495907

[111] Xu R Shimizu F Hovinga K Beal K Karimi S Droms L . Molecular and clinical effects of notch inhibition in glioma patients: a phase 0/I trial. Clin Cancer Res. (2016) 22:4786–96. doi: 10.1158/1078-0432.ccr-16-0048 27154916 PMC5050072

[112] Pan E Supko JG Kaley TJ Butowski NA Cloughesy T Jung J . Phase I study of RO4929097 with bevacizumab in patients with recurrent malignant glioma. J Neuro-Oncol. (2016) 130:571–9. doi: 10.1007/s11060-016-2263-1 27826680 PMC5571739

[113] Peereboom DM Ye X Mikkelsen T Lesser GJ Lieberman FS Robins HI . A phase II and pharmacodynamic trial of RO4929097 for patients with recurrent/progressive glioblastoma. Neurosurgery. (2021) 88:246–51. doi: 10.1093/neuros/nyaa412_s080 PMC7919338 33027815

[114] Rahman A Lozada-Martinez ID Rahman S Ataullah AHM Munoz-Baez K Moscote-Salazar LR . Letter: A phase II and pharmacodynamic trial of RO4929097 for patients with recurrent/progressive glioblastoma. Neurosurgery. (2021) 89:E78–9. doi: 10.1093/neuros/nyab097 33822209

[115] Fabbro D Bauer M Murone M Lehal R . Notch inhibition in cancer: challenges and opportunities. Chimia (Aarau). (2020) 74:779–83. doi: 10.2533/chimia.2020.779 33115560

[116] Weber D Lehal R Frismantas V Bourquin J Bauer M Murone M . Pharmacological activity of CB‐103 in haematological malignancies–an oral pan‐Notch inhibitor with a novel mode of action. Hematological Oncol. (2017) 35:46. doi: 10.1002/hon.2437_28

[117] Wu Y Cain-Hom C Choy L Hagenbeek TJ de Leon GP Chen Y . Therapeutic antibody targeting of individual Notch receptors. Nature. (2010) 464:1052–7. doi: 10.1038/nature08878 20393564

[118] Floyd DH Kefas B Seleverstov O Mykhaylyk O Dominguez C Comeau L . Alpha-secretase inhibition reduces human glioblastoma stem cell growth *in vitro* and *in vivo* by inhibiting Notch. Neuro Oncol. (2012) 14:1215–26. doi: 10.1093/neuonc/nos157 22962413 PMC3452340

[119] Li H Wu Y Chen Y Lv J Qu C Mei T . Overcoming temozolomide resistance in glioma: recent advances and mechanistic insights. Acta Neuropathol Commun. (2025) 13:126. doi: 10.1186/s40478-025-02046-4 40468460 PMC12139195

[120] Andersson ER Lendahl U . Therapeutic modulation of Notch signalling—are we there yet? Nat Rev Drug Discov. (2014) 13:357–78. 10.1038/nrd4252 24781550

[121] Yahyanejad S Granton PV Hoeben A Vooijs MA . Benefits, limitations and opportunities of NOTCH inhibitors for treatment of glioma. Trans Cancer Res. (2016) 5. doi: 10.21037/tcr.2016.07.26

[122] Eckel-Passow JE Lachance DH Molinaro AM Walsh KM Decker PA Sicotte H . Glioma groups based on 1p/19q, IDH, and TERT promoter mutations in tumors. N Engl J Med. (2015) 372:2499–508. doi: 10.1056/nejmoa1407279 26061753 PMC4489704

[123] Brandes AA Franceschi E Tosoni A Blatt V Pession A Tallini G . MGMT promoter methylation status can predict the incidence and outcome of pseudoprogression after concomitant radiochemotherapy in newly diagnosed glioblastoma patients. J Clin Oncol. (2008) 26:2192–7. doi: 10.1200/jco.2007.14.8163 18445844

[124] Demuth T Rennert JL Hoelzinger DB Reavie LB Nakada M Beaudry C . Glioma cells on the run–the migratory transcriptome of 10 human glioma cell lines. BMC Genomics. (2008) 9:54. doi: 10.1186/1471-2164-9-54 18230158 PMC2275271

[125] Yang Y Li S To KK Zhu S Wang F Fu L . Tumor-associated macrophages remodel the suppressive tumor immune microenvironment and targeted therapy for immunotherapy. J Exp Clin Cancer Res. (2025) 44:145. doi: 10.1186/s13046-025-03377-9 40380196 PMC12083052

[126] Deftos ML Huang E Ojala EW Forbush KA Bevan MJ . Notch1 signaling promotes the maturation of CD4 and CD8 SP thymocytes. Immunity. (2000) 13:73–84. doi: 10.1016/s1074-7613(00)00009-1 10933396 PMC2780426

[127] Reizis B Leder P . Direct induction of T lymphocyte-specific gene expression by the mammalian Notch signaling pathway. Genes Dev. (2002) 16:295–300. doi: 10.1101/gad.960702 11825871 PMC155334

[128] Andaloussi AE Han Y Lesniak MS . Progression of intracranial glioma disrupts thymic homeostasis and induces T-cell apoptosis *in vivo*. Cancer Immunol Immunother. (2008) 57:1807–16. doi: 10.1007/s00262-008-0508-3 18392618 PMC11030257

[129] Rana M Liou KC Thakur A Nepali K Liou JP . Advancing glioblastoma therapy: learning from the past and innovations for the future. Cancer Lett. (2025) 617:217601. doi: 10.1016/j.canlet.2025.217601 40037502

[130] Gursel DB Berry N Boockvar JA . The contribution of Notch signaling to glioblastoma via activation of cancer stem cell self-renewal: the role of the endothelial network. Neurosurgery. (2012) 70:N19–21. doi: 10.1227/01.neu.0000410937.38828.6f 22251985

[131] Wang M Yu F Zhang Y Li P . Novel insights into Notch signaling in tumor immunity: potential targets for cancer immunotherapy. Front Immunol. (2024) 15:1352484. doi: 10.3389/fimmu.2024.1352484 38444855 PMC10912471

[132] Chow RD Guzman CD Wang G Schmidt F Youngblood MW Ye L . AAV-mediated direct *in vivo* CRISPR screen identifies functional suppressors in glioblastoma. Nat Neurosci. (2017) 20:1329–41. doi: 10.1038/nn.4620 28805815 PMC5614841

[133] Lin Y Zhao JL Zheng QJ Jiang X Tian J Liang SQ . Notch signaling modulates macrophage polarization and phagocytosis through direct suppression of signal regulatory protein alpha expression. Front Immunol. (2018) 9:1744. doi: 10.3389/fimmu.2018.01744 30105024 PMC6077186

[134] Wang EJ Chen JS Jain S Morshed RA Haddad AF Gill S . Immunotherapy resistance in glioblastoma. Front Genet. (2021) 12:750675. doi: 10.3389/fgene.2021.750675 34976006 PMC8718605

[135] Kanamori M Kawaguchi T Nigro JM Feuerstein BG Berger MS Miele L . Contribution of Notch signaling activation to human glioblastoma multiforme. J Neurosurg. (2007) 106:417–27. doi: 10.3171/jns.2007.106.3.417 17367064

[136] El Hindy N Keyvani K Pagenstecher A Dammann P Sandalcioglu IE Sure U . Implications of Dll4-Notch signaling activation in primary glioblastoma multiforme. Neuro Oncol. (2013) 15:1366–78. doi: 10.1093/neuonc/not071 23787764 PMC3779034

[137] Roesch S Rapp C Dettling S Herold-Mende C . When immune cells turn bad-tumor-associated microglia/macrophages in glioma. Int J Mol Sci. (2018) 19. doi: 10.3390/ijms19020436 29389898 PMC5855658

[138] Hambardzumyan D Bergers G . Glioblastoma: defining tumor niches. Trends Cancer. (2015) 1:252–65. doi: 10.1016/j.trecan.2015.10.009 27088132 PMC4831073

[139] Yabo YA Moreno-Sanchez PM Pires-Afonso Y Kaoma T Nosirov B Scafidi A . Glioblastoma-instructed microglia transition to heterogeneous phenotypic states with phagocytic and dendritic cell-like features in patient tumors and patient-derived orthotopic xenografts. Genome Med. (2024) 16:51. doi: 10.1186/s13073-024-01321-8 38566128 PMC10988817

[140] Alterman RL Stanley ER . Colony stimulating factor-1 expression in human glioma. Mol Chem Neuropathol. (1994) 21:177–88. doi: 10.1007/bf02815350 8086034

[141] Zhai H Heppner FL Tsirka SE . Microglia/macrophages promote glioma progression. Glia. (2011) 59:472–85. doi: 10.1002/glia.21117 PMC3080032 21264953

[142] Mohammadi Y Nourbakhsh M . A mini review on the multifaceted role of TGF-β in metastasis progression: Molecular mechanisms and novel therapeutic strategies. Acta Biochim Iranica. (2024) 2:125–31.

[143] Espinoza FI Tankov S Chliate S Pereira Couto J Marinari E Vermeil T . Targeting HIF-2alpha in glioblastoma reshapes the immune infiltrate and enhances response to immune checkpoint blockade. Cell Mol Life Sci. (2025) 82:119. doi: 10.1007/s00018-025-05642-8 40095115 PMC11914682

[144] Watowich MB Gilbert MR Larion M . T cell exhaustion in malignant gliomas. Trends Cancer. (2023) 9:270–92. doi: 10.1016/j.trecan.2022.12.008 36681605 PMC10038906

[145] Moujaess E Haddad FG Eid R Kourie HR . The emerging use of immune checkpoint blockade in the adjuvant setting for solid tumors: a review. Immunotherapy. (2019) 11:1409–22. doi: 10.2217/imt-2019-0087 31621445

[146] Keshavarz Sadegh R Saleki K Rezaei N . Immune checkpoint inhibitor (ICI) therapy in central nervous system cancers: State-of-the-art and future outlook. Int Immunopharmacol. (2025) 159:114837. doi: 10.1016/j.intimp.2025.114837 40394797

[147] Agosti E Zeppieri M De Maria L Tedeschi C Fontanella MM Panciani PP . Glioblastoma immunotherapy: A systematic review of the present strategies and prospects for advancements. Int J Mol Sci. (2023) 24. doi: 10.3390/ijms242015037 37894718 PMC10606063

[148] Wang D Zhang J Bu C Liu G Guo G Zhang Z . Dynamics of tumor in situ fluid circulating tumor DNA in recurrent glioblastomas forecasts treatment efficacy of immune checkpoint blockade coupled with low-dose bevacizumab. J Cancer Res Clin Oncol. (2024) 150:466. doi: 10.1007/s00432-024-05997-8 39422764 PMC11489198

[149] Poonaki E Kahlert UD Meuth SG Gorji A . The role of the ZEB1-neuroinflammation axis in CNS disorders. J Neuroinflamm. (2022) 19:275. doi: 10.1186/s12974-022-02636-2 36402997 PMC9675144

[150] Zheng C Wu H Jin S Li D Tan S Zhu X . Roles of Myc-associated zinc finger protein in malignant tumors. Asia Pac J Clin Oncol. (2022) 18:506–14. doi: 10.1111/ajco.13748 35098656

[151] Zhu Q Liu Z Cheng X Liang W Wang H Li P . ZNF480 influences the prognosis, pathogenesis, and immune microenvironment in patients with lower-grade glioma. Heliyon. (2023) 9:e18185. doi: 10.1016/j.heliyon.2023.e18185 37519705 PMC10372659

[152] Liang W Hu C Zhu Q Cheng X Gao S Liu Z . Exploring the relationship between abnormally high expression of NUP205 and the clinicopathological characteristics, immune microenvironment, and prognostic value of lower-grade glioma. Front Oncol. (2023) 13:1007198. doi: 10.3389/fonc.2023.1007198 37284202 PMC10240054

[153] Asad M Inocencio J Mitrasinovic S Aoki M Baker CE Hormigo A . TIGIT expression dictates the immunosuppressive reprogramming of myeloid cells in glioblastoma. Neuro Oncol. (2026) 28:1426–41. doi: 10.1093/neuonc/noag044 41741381 PMC13231175

[154] Mair MJ Kiesel B Feldmann K Widhalm G Dieckmann K Wohrer A . LAG-3 expression in the inflammatory microenvironment of glioma. J Neuro-Oncol. (2021) 152:533–9. doi: 10.1007/s11060-021-03721-x 33651248 PMC8084780

[155] Woo SR Turnis ME Goldberg MV Bankoti J Selby M Nirschl CJ . Immune inhibitory molecules LAG-3 and PD-1 synergistically regulate T-cell function to promote tumoral immune escape. Cancer Res. (2012) 72:917–27. doi: 10.1158/0008-5472.can-11-1620 22186141 PMC3288154

[156] Andrews LP Marciscano AE Drake CG Vignali DA . LAG3 (CD223) as a cancer immunotherapy target. Immunol Rev. (2017) 276:80–96. doi: 10.1111/imr.12519 PMC5338468 28258692

[157] Wang J Sanmamed MF Datar I Su TT Ji L Sun J . Fibrinogen-like protein 1 is a major immune inhibitory ligand of LAG-3. Cell. (2019) 176:334–47:e312. doi: 10.1016/j.cell.2018.11.010 30580966 PMC6365968

[158] Huang H Li C You H Zhang Z Lin Q Yang J . Radiation-induced glioblastoma stem cell-mediated T cell exhaustion via EGR1-Gal3-LAG3 axis in glioblastoma. Cancer Lett. (2026) 636:218125. doi: 10.1016/j.canlet.2025.218125 41213464

[159] Das M Zhu C Kuchroo VK . Tim-3 and its role in regulating anti-tumor immunity. Immunol Rev. (2017) 276:97–111. doi: 10.1111/imr.12520 28258697 PMC5512889

[160] Huang YH Zhu C Kondo Y Anderson AC Gandhi A Russell A . Author correction: CEACAM1 regulates TIM-3-mediated tolerance and exhaustion. Nature. (2024) 626:E19. doi: 10.1038/s41586-024-07164-y 38336833

[161] Ahmady F Sharma A Achuthan AA Kannourakis G Luwor RB . The role of TIM-3 in glioblastoma progression. Cells. (2025) 14. doi: 10.3390/cells14050346 40072074 PMC11899008

[162] Ahmady-Nield F Carnie BM Anderson GE Achari E Sharma A Achuthan AA . The signaling networks of TIM-3, TGF-β, and STING in glioblastoma. Cells. (2026) 15:991. doi: 10.3390/cells15110991 42274584 PMC13257219

[163] de Mingo Pulido A Hanggi K Celias DP Gardner A Li J Batista-Bittencourt B . The inhibitory receptor TIM-3 limits activation of the cGAS-STING pathway in intra-tumoral dendritic cells by suppressing extracellular DNA uptake. Immunity. (2021) 54:1154–67:e1157. doi: 10.1016/j.immuni.2021.04.019 33979578 PMC8192496

[164] Yan Z Wang C Wu J Wang J Ma T . TIM-3 teams up with PD-1 in cancer immunotherapy: mechanisms and perspectives. Mol BioMed. (2025) 6:27. doi: 10.1186/s43556-025-00267-6 40332725 PMC12058639

[165] Parmigiani E Ivanek R Rolando C Hafen K Turchinovich G Lehmann FM . Interferon-gamma resistance and immune evasion in glioma develop via Notch-regulated co-evolution of malignant and immune cells. Dev Cell. (2022) 57:1847–65:e1849. doi: 10.1016/j.devcel.2022.06.006 35803280

[166] Collu GM Hidalgo-Sastre A Brennan K . Wnt-Notch signalling crosstalk in development and disease. Cell Mol Life Sci. (2014) 71:3553–67. doi: 10.1007/s00018-014-1644-x 24942883 PMC11113451

[167] Liu G Yan T Li X Sun J Zhang B Wang H . Daam1 activates RhoA to regulate Wnt5a-induced glioblastoma cell invasion. Oncol Rep. (2018) 39:465–72. 10.3892/or.2017.6124 PMC5783613 29207169

[168] Feng Y Wang Y Guo K Feng J Shao C Pan M . The value of WNT5A as prognostic and immunological biomarker in pan-cancer. Ann Transl Med. (2022) 10:466. doi: 10.21037/atm-22-1317 35571400 PMC9096401

[169] Ishikawa T Ogura Y Tanaka K Nagashima H Sasayama T Endo M . Ror1 is expressed inducibly by Notch and hypoxia signaling and regulates stem cell-like property of glioblastoma cells. Cancer Sci. (2023) 114:561–73. doi: 10.1111/cas.15630 PMC9899608 36314076

[170] Liu YT Sun ZJ . Turning cold tumors into hot tumors by improving T-cell infiltration. Theranostics. (2021) 11:5365–86. doi: 10.7150/thno.58390 33859752 PMC8039952

[171] Zhao M Chen S . The role of VANGL2 in glioma oncogenesis and progression: insights into expression profiles and prognostic relevance. Front Oncol. (2024) 14:1527226. doi: 10.2174/1386207326666230502103726 39871948 PMC11770008

[172] Schreck KC Taylor P Marchionni L Gopalakrishnan V Bar EE Gaiano N . The Notch target Hes1 directly modulates Gli1 expression and Hedgehog signaling: a potential mechanism of therapeutic resistance. Clin Cancer Res. (2010) 16:1660–70. doi: 10.1158/1078-0432.CCR-10-1624 PMC3059501 21169257

[173] Felli MP Maroder M Mitsiadis TA Campese AF Bellavia D Vacca A . Expression pattern of notch1, 2 and 3 and Jagged1 and 2 in lymphoid and stromal thymus components: distinct ligand-receptor interactions in intrathymic T cell development. Int Immunol. (1999) 11:1017–25. doi: 10.1093/intimm/11.7.1017 10383933

[174] Grivennikov SI Greten FR Karin M . Immunity, inflammation, and cancer. Cell. (2010) 140:883–99. doi: 10.1016/j.cell.2010.01.025 20303878 PMC2866629

[175] Burban A Sharanek A Hernandez-Corchado A Najafabadi HS Soleimani VD Jahani-Asl A . Targeting glioblastoma with a brain-penetrant drug that impairs brain tumor stem cells via NLE1-Notch1 complex. Stem Cell Rep. (2024) 19:1534–47. doi: 10.1016/j.stemcr.2024.09.007 39423824 PMC11589194

[176] Tang J Amin MA Campian JL . Glioblastoma stem cells at the nexus of tumor heterogeneity, immune evasion, and therapeutic resistance. Cells. (2025) 14. doi: 10.20944/preprints202503.1215.v1 PMC12025403 40277888

[177] Ramar V Guo S Hudson B Liu M . Progress in glioma stem cell research. Cancers (Basel). (2023) 16. doi: 10.3390/cancers16010102 38201528 PMC10778204

[178] MacLeod G Molaei F Haider S Almeida MP Lin S Kushida M . Fitness screens map state-specific glioblastoma stem cell vulnerabilities. Cancer Res. (2024) 84:3967–83. doi: 10.1158/0008-5472.can-23-4024 39186687

[179] Jin X Kim SH Jeon HM Beck S Sohn YW Yin J . Interferon regulatory factor 7 regulates glioma stem cells via interleukin-6 and Notch signalling. Brain. (2012) 135:1055–69. doi: 10.1093/brain/aws028 22434214

[180] Chang WH Lai AG . Aberrations in Notch-Hedgehog signalling reveal cancer stem cells harbouring conserved oncogenic properties associated with hypoxia and immunoevasion. Br J Cancer. (2019) 121:666–78. doi: 10.1038/s41416-019-0572-9 31523055 PMC6889439

[181] Haapa-Paananen S Kiviluoto S Waltari M Puputti M Mpindi JP Kohonen P . HES6 gene is selectively overexpressed in glioma and represents an important transcriptional regulator of glioma proliferation. Oncogene. (2012) 31:1299–310. doi: 10.1038/onc.2011.316 21785461

[182] Sun Y Liu P Lv D Yang D Qiao S Liu J . Pan-cancer analysis reveals dual roles of HES6 in cancer immunity and prognosis. Discov Oncol. (2025) 16:1110. doi: 10.1007/s12672-025-02852-1 40517338 PMC12167741

[183] Schreck KC Taylor P Marchionni L Gopalakrishnan V Bar EE Gaiano N . The Notch target Hes1 directly modulates Gli1 expression and Hedgehog signaling: a potential mechanism of therapeutic resistance. Clin Cancer Res. (2010) 16:6060–70. doi: 10.1158/1078-0432.ccr-10-1624 21169257 PMC3059501

[184] Gampala S Yang JY . Hedgehog pathway inhibitors against tumor microenvironment. Cells. (2021) 10. doi: 10.3390/cells10113135 34831357 PMC8619966

[185] Collinson R Tanos B . Primary cilia and cancer: a tale of many faces. Oncogene. (2025) 44:1551–66. doi: 10.1038/s41388-025-03416-x 40301543 PMC12095056

[186] Liu H Kiseleva AA Golemis EA . Ciliary signalling in cancer. Nat Rev Cancer. (2018) 18:511–24. doi: 10.1038/s41568-018-0023-6 29802351 PMC6448793

[187] Sarkisian MR Semple-Rowland SL . Emerging roles of primary cilia in glioma. Front Cell Neurosci. (2019) 13:55. doi: 10.3389/fncel.2019.00055 30842728 PMC6391589

[188] Uwishema O Shariff S Wojtara M Chakik JAE Ghosh S Obamiro K . Stem cell therapies and glioma stem cells in glioblastoma: a systematic review of current challenges and research directions. Int J Emerg Med. (2025) 18:144. doi: 10.1186/s12245-025-00921-4 40770790 PMC12326703

[189] Yi L Zhou X Li T Liu P Hai L Tong L . Notch1 signaling pathway promotes invasion, self-renewal and growth of glioma initiating cells via modulating chemokine system CXCL12/CXCR4. J Exp Clin Cancer Res. (2019) 38:339. doi: 10.1186/s13046-019-1319-4 31382985 PMC6683584

[190] Zhang T Zhang Q He X Lu Y Shao A Sun X . Identification of key molecular pathways and associated genes as targets to overcome radiotherapy resistance using a combination of radiotherapy and immunotherapy in glioma patients. Int J Mol Sci. (2024) 25. doi: 10.3390/ijms25053076 38474320 PMC10931693

[191] Hao B Chen X Cao Y . Yes-associated protein 1 promotes the metastasis of U251 glioma cells by upregulating Jagged-1 expression and activating the Notch signal pathway. Exp Ther Med. (2018) 16:1411–6. doi: 10.3892/etm.2018.6322 30112068 PMC6090459

[192] Chockalingam S Ghosh SS . Amelioration of cancer stem cells in macrophage colony stimulating factor-expressing U87MG-human glioblastoma upon 5-fluorouracil therapy. PloS One. (2013) 8:e83877. doi: 10.1371/journal.pone.0083877 24391839 PMC3877109

[193] Lino MM Merlo A Boulay J-L . Notch signaling in glioblastoma: a developmental drug target? BMC Med. (2010) 8:72. doi: 10.1186/1741-7015-8-72 21078177 PMC2996337

[194] Wang J Wakeman TP Lathia JD Hjelmeland AB Wang X-F White RR . Notch promotes radioresistance of glioma stem cells. Stem Cells. (2010) 28:17–28. doi: 10.1002/stem.261 PMC2825687 19921751

[195] Dantas-Barbosa C Bergthold G Daudigeos-Dubus E Blockus H Boylan JF Ferreira C . Inhibition of the NOTCH pathway using gamma-secretase inhibitor RO4929097 has limited antitumor activity in established glial tumors. Anticancer Drugs. (2015) 26:272–83. doi: 10.1097/cad.0000000000000190 25486598

[196] Su P Li O Ke K Jiang Z Wu J Wang Y . Targeting tumor-associated macrophages: Critical players in tumor progression and therapeutic strategies (Review). Int J Oncol. (2024) 64. doi: 10.3892/ijo.2024.5648 38695252 PMC11087038

[197] Lu C Kang T Zhang J Yang K Liu Y Song K . Combined targeting of glioblastoma stem cells of different cellular states disrupts malignant progression. Nat Commun. (2025) 16:2974. doi: 10.1038/s41467-025-58366-5 40140646 PMC11947120

[198] Sun J Gong X Purow B Zhao Z . Uncovering microRNA and transcription factor mediated regulatory networks in glioblastoma. PloS Comput Biol. (2012) 8:e1002488. doi: 10.1371/journal.pcbi.1002488 22829753 PMC3400583

[199] Makowska M Smolarz B Romanowicz H . microRNAs (miRNAs) in glioblastoma multiforme (GBM)-recent literature review. Int J Mol Sci. (2023) 24. doi: 10.3390/ijms24043521 36834933 PMC9965735

[200] Wu Z Wu Y Tian Y Sun X Liu J Ren H . Differential effects of miR-34c-3p and miR-34c-5p on the proliferation, apoptosis and invasion of glioma cells. Oncol Lett. (2013) 6:1447–52. doi: 10.3892/ol.2013.1579 24179539 PMC3813786

[201] Wong HA Fatimy RE Onodera C Wei Z Yi M Mohan A . The Cancer Genome Atlas analysis predicts microRNA for targeting cancer growth and vascularization in glioblastoma. Mol Ther. (2015) 23:1234–47. doi: 10.1038/mt.2015.72 25903473 PMC4817797

[202] Tang B Wu W Wei X Li Y Ren G Fan W . Activation of glioma cells generates immune tolerant NKT cells. J Biol Chem. (2014) 289:34595–600. doi: 10.1074/jbc.m114.614503 25342742 PMC4263866

[203] Khwaja SS Cai C Badiyan SN Wang X Huang J . The immune-related microRNA miR-146b is upregulated in glioblastoma recurrence. Oncotarget. (2018) 9:29036–46. doi: 10.18632/oncotarget.25528 30018734 PMC6044384

[204] Mirzaei S Ahangari F Faramarzi F Khoshnazar SM Khormizi FZ Aghagolzadeh M . MicroRNA-146 family: Molecular insights into their role in regulation of signaling pathways in glioma progression. Pathol Res Pract. (2024) 264:155707. doi: 10.1016/j.prp.2024.155707 39536541

[205] Qian Z Zhou S Zhou Z Yang X Que S Lan J . miR-146b-5p suppresses glioblastoma cell resistance to temozolomide through targeting TRAF6. Oncol Rep. (2017) 38:2941–50. doi: 10.3892/or.2017.5970 29048680

[206] Gao C Wei J Tang T Huang Z . Role of microRNA-33a in malignant cells. Oncol Lett. (2020) 20:2537–56. doi: 10.3892/ol.2020.11835 PMC7399786 32782572

[207] Wang H Sun T Hu J Zhang R Rao Y Wang S . miR-33a promotes glioma-initiating cell self-renewal via PKA and NOTCH pathways. J Clin Invest. (2014) 124:4489–502. doi: 10.1172/jci75284 25202981 PMC4191031

[208] Marino S Menna G Di Bonaventura R Lisi L Mattogno P Figa F . The extracellular matrix in glioblastomas: A glance at its structural modifications in shaping the tumoral microenvironment-a systematic review. Cancers (Basel). (2023) 15. doi: 10.3390/cancers15061879 36980765 PMC10046791

[209] Xiao W Sohrabi A Seidlits SK . Integrating the glioblastoma microenvironment into engineered experimental models. Future Sci OA. (2017) 3:FSO189. doi: 10.4155/fsoa-2016-0094 28883992 PMC5583655

[210] Hao Y Zhou X Li Y Li B Cheng L . The CD47-SIRPalpha axis is a promising target for cancer immunotherapies. Int Immunopharmacol. (2023) 120:110255. doi: 10.1016/j.intimp.2023.110255 37187126

[211] Ma D Liu S Lal B Wei S Wang S Zhan D . Extracellular matrix protein tenascin C increases phagocytosis mediated by CD47 loss of function in glioblastoma. Cancer Res. (2019) 79:2697–708. doi: 10.1158/0008-5472.can-18-3125 30898840 PMC8218246

[212] Li Y Hu Y Liu C Wang Q Han X Han Y . Human fibulin-3 protein variant expresses anti-cancer effects in the malignant glioma extracellular compartment in intracranial xenograft models. Oncotarget. (2017) 8:106311–23. doi: 10.18632/oncotarget.22344 29290950 PMC5739735

[213] Ke C Luo JR Cen ZW Li Y Cai HP Wang J . Dual antivascular function of human fibulin-3 variant, a potential new drug discovery strategy for glioblastoma. Cancer Sci. (2020) 111:940–50. doi: 10.1111/cas.14300 31922633 PMC7060460

[214] Uddin MS Mamun AA Alghamdi BS Tewari D Jeandet P Sarwar MS . Epigenetics of glioblastoma multiforme: From molecular mechanisms to therapeutic approaches. Semin Cancer Biol. (2022) 83:100–20. doi: 10.1016/j.semcancer.2020.12.015 33370605

[215] Shahani A Slika H Elbeltagy A Lee A Peters C Dotson T . The epigenetic mechanisms involved in the treatment resistance of glioblastoma. Cancer Drug Resist. (2025) 8:12. doi: 10.20517/cdr.2024.157 40201311 PMC11977385

[216] Xie J Qiao L Deng G Liang N Xing L Zhang J . PCGF1 is a prognostic biomarker and correlates with tumor immunity in gliomas. Ann Transl Med. (2022) 10:227. doi: 10.21037/atm-22-198 35280367 PMC8908126

[217] Schapira M Tyers M Torrent M Arrowsmith CH . WD40 repeat domain proteins: a novel target class? Nat Rev Drug Discov. (2017) 16:773–86. doi: 10.1038/nrd.2017.179 29026209 PMC5975957

[218] Gurung R Om D Pun R Hyun S Shin D . Recent progress in modulation of WD40-repeat domain 5 protein (WDR5): Inhibitors and degraders. Cancers (Basel). (2023) 15. doi: 10.3390/cancers15153910 37568727 PMC10417795

[219] Cheng X Liu Z Chang H Liang W Li P Gao Y . WD repeat domain 76 predicts poor prognosis in lower grade glioma and provides an original target for immunotherapy. Eur J Med Res. (2024) 29:13. doi: 10.1186/s40001-023-01605-6 38173030 PMC10763342

[220] Geng W Liang W Fan Y Ye Z Zhang L . Overexpression of CCDC34 in colorectal cancer and its involvement in tumor growth, apoptosis and invasion. Mol Med Rep. (2018) 17:465–73. 10.3892/mmr.2017.7860 PMC5780142 29115580

[221] Shen H Li J Jia Y Liu F . The oncogenic role of CCDC34 in lower-grade gliomas: prognostic significance and therapeutic potential. J Neuro-Oncol. (2025) 175:71–89. doi: 10.1007/s11060-025-05090-1 40522566 PMC12367944

[222] Turcan S Rohle D Goenka A Walsh LA Fang F Yilmaz E . IDH1 mutation is sufficient to establish the glioma hypermethylator phenotype. Nature. (2012) 483:479–83. doi: 10.1038/nature10866 22343889 PMC3351699

[223] Wang H Zheng T Xu D Sun C Huang D Liu X . Targeting DLL3: Innovative strategies for tumor treatment. Pharmaceutics. (2025) 17. doi: 10.3390/pharmaceutics17040520 40284515 PMC12030079

[224] Cao Y Liu B Cai L Li Y Huang Y Zhou Y . G9a promotes immune suppression by targeting the Fbxw7/Notch pathway in glioma stem cells. CNS Neurosci Ther. (2023) 29:2508–21. doi: 10.1111/cns.14191 36971192 PMC10401078

[225] Hall K Ran S . Regulation of tumor angiogenesis by the local environment. Front Biosci (Landmark Ed). (2010) 15:195–212. doi: 10.2741/3615 20036815

[226] Cho A McKelvey KJ Lee A Hudson AL . The intertwined fates of inflammation and coagulation in glioma. Mamm Genome. (2018) 29:806–19. doi: 10.1007/s00335-018-9761-8 30062485

[227] Huang T Sun F Gao K Wang Y Zhu G Chen F . The role of peripheral inflammatory markers and coagulation factors in patients with central nervous system (CNS) immune disease and glioma. World Neurosurg. (2024) 188:e177–93. doi: 10.1016/j.wneu.2024.05.080 38763458

[228] Zhu H Yu X Zhang S Shu K . Targeting the complement pathway in malignant glioma microenvironments. Front Cell Dev Biol. (2021) 9:657472. doi: 10.3389/fcell.2021.657472 33869223 PMC8047198

[229] Yang J Shen L Yang J Qu Y Gong C Zhou F . Complement and coagulation cascades are associated with prognosis and the immune microenvironment of lower-grade glioma. Transl Cancer Res. (2024) 13:112–36. doi: 10.21037/tcr-23-906 38410234 PMC10894340

[230] You WK Schuetz TJ Lee SH . Targeting the DLL/Notch signaling pathway in cancer: Challenges and advances in clinical development. Mol Cancer Ther. (2023) 22:3–11. doi: 10.1158/1535-7163.MCT-22-0243 PMC9808372 36223541

[231] Wang K Zhang Q Li D Ching K Zhang C Zheng X . PEST domain mutations in Notch receptors comprise an oncogenic driver segment in triple-negative breast cancer sensitive to a gamma-secretase inhibitor. Clin Cancer Res. (2015) 21:1487–96. doi: 10.1158/1078-0432.ccr-14-1348 25564152

[232] Parmigiani E Giachino C . Genetic inactivation of Notch1 synergizes with loss of Trp53 to induce tumor formation in the adult mouse forebrain. Cancers (Basel). (2022) 14. doi: 10.3390/cancers14215409 36358826 PMC9655454

[233] Sivasankaran B Degen M Ghaffari A Hegi ME Hamou MF Ionescu MC . Tenascin-C is a novel RBPJkappa-induced target gene for Notch signaling in gliomas. Cancer Res. (2009) 69:458–65. doi: 10.1158/0008-5472.can-08-2610 19147558

[234] Long J Wang D Yang X Wang A Lin Y Zheng M . Identification of NOTCH4 mutation as a response biomarker for immune checkpoint inhibitor therapy. BMC Med. (2021) 19:154. doi: 10.1186/s12916-021-02031-3 34284787 PMC8293505

[235] Minter LM Turley DM Das P Shin HM Joshi I Lawlor RG . Inhibitors of gamma-secretase block *in vivo* and *in vitro* T helper type 1 polarization by preventing Notch upregulation of Tbx21. Nat Immunol. (2005) 6:680–8. doi: 10.1038/ni1209x 15991363

[236] Morsut L Roybal KT Xiong X Gordley RM Coyle SM Thomson M . Engineering customized cell sensing and response behaviors using synthetic Notch receptors. Cell. (2016) 164:780–91. doi: 10.1016/j.cell.2016.01.012 26830878 PMC4752866

[237] Roybal KT Rupp LJ Morsut L Walker WJ McNally KA Park JS . Precision tumor recognition by T cells with combinatorial antigen-sensing circuits. Cell. (2016) 164:770–9. doi: 10.1016/j.cell.2016.01.011 26830879 PMC4752902

[238] Xiu M Zeng X Shan R Wen W Li J Wan R . Targeting Notch4 in cancer: Molecular mechanisms and therapeutic perspectives. Cancer Manag Res. (2021) 13:7033–45. doi: 10.2147/cmar.s315511 34526819 PMC8436177

[239] Sun R Kim AH . The multifaceted mechanisms of malignant glioblastoma progression and clinical implications. Cancer Metastasis Rev. (2022) 41:871–98. doi: 10.1007/s10555-022-10051-5 35920986 PMC9758111

[240] Watson SS Zomer A Fournier N Lourenco J Quadroni M Chryplewicz A . Fibrotic response to anti-CSF-1R therapy potentiates glioblastoma recurrence. Cancer Cell. (2024) 42:1507–27:e1511. doi: 10.1016/j.ccell.2024.08.012 39255775

[241] Wu J Wang N . Current progress of anti-PD-1/PDL1 immunotherapy for glioblastoma (review). Mol Med Rep. (2024) 30. doi: 10.3892/mmr.2024.13344 39364736 PMC11462401

[242] Hu B Wang Q Wang YA Hua S Sauve CG Ong D . Epigenetic activation of WNT5A drives glioblastoma stem cell differentiation and invasive growth. Cell. (2016) 167:1281–95:e1218. doi: 10.1016/j.cell.2016.10.039 27863244 PMC5320931

[243] Li Z Huang Q Chen H Lin Z Zhao M Jiang Z . Interferon regulatory factor 7 promoted glioblastoma progression and stemness by modulating IL-6 expression in microglia. J Cancer. (2017) 8:207–19. doi: 10.7150/jca.16415 28243325 PMC5327370

[244] Mohiuddin E Wakimoto H . Extracellular matrix in glioblastoma: Opportunities for emerging therapeutic approaches. Am J Cancer Res. (2021) 11:3742–54. PMC8414390 34522446

[245] Zhang M Ye G Li J Wang Y . Recent advance in molecular angiogenesis in glioblastoma: The challenge and hope for anti-angiogenic therapy. Brain Tumor Pathol. (2015) 32:229–36. doi: 10.1007/s10014-015-0233-5 26437643

[246] Towner RA Zalles M Saunders D Smith N . Novel approaches to combat chemoresistance against glioblastomas. Cancer Drug Resist. (2020) 3:686–98. doi: 10.20517/cdr.2020.38 35582224 PMC8992560

[247] Mout R Jing R Tanaka-Yano M Egan ED Eisenach H Kononov MA . Design of soluble Notch agonists that drive T cell development and boost immunity. Cell. (2025) 188:5980–94.e28. doi: 10.1016/j.cell.2025.07.009 40752493 PMC12327808

[248] Herrera-Rios D Li G Khan D Tsiampali J Nickel AC Aretz P . A computational guided, functional validation of a novel therapeutic antibody proposes Notch signaling as a clinical relevant and druggable target in glioma. Sci Rep. (2020) 10:16218. doi: 10.1038/s41598-020-72480-y 33004830 PMC7531005

